# Identification of food deprivation in salmonids using gill biomarkers

**DOI:** 10.1093/conphys/coaf088

**Published:** 2025-12-19

**Authors:** William S Bugg, Arash Akbarzadeh, Tobi Ming, Angela D Schulze, Emiliano Di Cicco, Emily Yungwirth, Jennifer Curtis, David A Patterson, William D P Duguid, Andrew W Bateman, Kristina M Miller

**Affiliations:** Pacific Salmon Foundation, 1385 West 8th Avenue, Vancouver, BC V6H 3V9, Canada; Department of Forest and Conservation Sciences, University of British Columbia, 2424 Main Mall, Vancouver, BC V6T 1Z4, Canada; Pacific Biological Station, Fisheries and Oceans Canada, 3190 Hammond Bay Road, Nanaimo, BC V9T 6N7, Canada; Department of Fisheries, Faculty of Marine Science and Technology, University of Hormozgan, KM 9 of Minab Road, Bandar Abbas, Iran; Pacific Biological Station, Fisheries and Oceans Canada, 3190 Hammond Bay Road, Nanaimo, BC V9T 6N7, Canada; Pacific Biological Station, Fisheries and Oceans Canada, 3190 Hammond Bay Road, Nanaimo, BC V9T 6N7, Canada; Pacific Salmon Foundation, 1385 West 8th Avenue, Vancouver, BC V6H 3V9, Canada; Cultus Lake Lab, Fisheries and Oceans Canada, 4222 Columbia Valley Road, Cultus Lake, BC V2R 5B6, Canada; Cooperative Resource Management Institute, School of Resource and Environmental Management, Simon Fraser University, 8888 University Drive, Burnaby, BC V5A 1S6, Canada; Cultus Lake Lab, Fisheries and Oceans Canada, 4222 Columbia Valley Road, Cultus Lake, BC V2R 5B6, Canada; Cooperative Resource Management Institute, School of Resource and Environmental Management, Simon Fraser University, 8888 University Drive, Burnaby, BC V5A 1S6, Canada; Cultus Lake Lab, Fisheries and Oceans Canada, 4222 Columbia Valley Road, Cultus Lake, BC V2R 5B6, Canada; Cooperative Resource Management Institute, School of Resource and Environmental Management, Simon Fraser University, 8888 University Drive, Burnaby, BC V5A 1S6, Canada; Pacific Salmon Foundation, 1385 West 8th Avenue, Vancouver, BC V6H 3V9, Canada; Department of Biology, University of Victoria, 3800 Finnerty Road, Victoria, BC V8P 5C2, Canada; Pacific Salmon Foundation, 1385 West 8th Avenue, Vancouver, BC V6H 3V9, Canada; Department of Forest and Conservation Sciences, University of British Columbia, 2424 Main Mall, Vancouver, BC V6T 1Z4, Canada; Pacific Biological Station, Fisheries and Oceans Canada, 3190 Hammond Bay Road, Nanaimo, BC V9T 6N7, Canada

**Keywords:** Energetic status, fit-chip, food deprivation, gene expression, Random forest, RNA-seq, salmon

## Abstract

Risk assessments have identified prey limitation as one of the strongest risk factors for juvenile salmon survival under climate change. In British Columbia, Canada, juvenile Chinook salmon (*Oncorhynchus tshawytscha*) may experience prolonged periods of food deprivation upon marine entry and during their first marine winter. We assessed the physiological and transcriptional consequences of food deprivation to discover and develop mRNA-based biomarkers for food deprivation in the gill of juvenile Chinook salmon. Gill and liver tissue were collected from juvenile Chinook salmon held at 16 or 8°C that were fed or food deprived for up to 56 days and during a 21-day refeeding period. Chinook salmon at 16 and 8°C were able to withstand food deprivation for periods of 35 and 56 days, respectively, with declines in body morphometrics, hepatosomatic index, insulin-like growth factor-1 and energy density observed in food-deprived individuals, followed by rapid recovery during refeeding. RNA-sequencing at the end of the food deprivation period revealed candidate biomarkers for food deprivation representing structural and functional components of the gill as well as metabolic processes like lipid storage and energy metabolism in the liver. Using the strongest 12 gill biomarkers paired with high-throughput qPCR and a random forest classification model, transcriptional signatures of food deprivation were detected within 14 to 28 days following food deprivation and persisted for at least 6 days following refeeding. These gill biomarkers can be non-lethally applied to wild juvenile salmon to answer long standing questions regarding food deprivation and the drivers of mortality during their early marine migration and overwintering.

## Introduction

Many Pacific salmon (*Oncorhynchus sp.*) populations along the eastern Pacific coast have suffered declines throughout the past half century (Irvine and Fukuwaka, 2011; Ohlberger *et al.*, 2018; Welch *et al.*, 2021). These declines have raised concerns of local extirpation for Pacific salmon themselves (Cosewic, 2018) and for the myriad species that rely on them as foundational prey species. Declines in many Pacific salmon populations appear to be driven by poor early marine survival (Beamish and Mahnken, 2001; Moss *et al.*, 2005; Duffy and Beauchamp, 2011; Woodson *et al.*, 2013; Graham, 2016; Duguid *et al.*, 2018; COSEWIC, 2020; Beamish, 2022), often associated with size selective processes that are critical in determining overall survival patterns (Beamish and Mahnken, 2001; Welch *et al.*, 2021). During this early marine period, food limitation is likely a key stressor (Chittenden *et al.*, 2010; Irvine and Akenhead, 2013), and interacts with other environmental stressors like pathogens and predation to limit survival (Miller *et al.*, 2014; Furey *et al.*, 2021; Bass *et al.*, 2022; Deeg *et al.*, 2022), triggering a variety of physiological and molecular responses across tissues (Esmaeili *et al.*, 2021; Esmaeili *et al.*, 2023). Furthermore, as climate change intensifies, evidence suggests that available habitat and prey abundance may be diminished either through direct effects or phenological mismatch (Abdul-Aziz *et al.*, 2011; Siegel, 2020), increasing the risks of food deprivation, leading to physiological increased metabolic demand, stress and mortality in Pacific salmon (Crozier *et al.*, 2021). Thus, even if food is potentially available in the environment, the impacts of other stressors may suppress feeding, reduce ability to feed or limit feeding opportunities, leading to food deprivation (McCormick *et al.*, 1998; Pankhurst *et al.*, 2008; Shuntov *et al.*, 2017; Shuntov *et al.*, 2019).

Juvenile Chinook salmon (*O. tshawytshca*) may be particularly vulnerable to food deprivation during their freshwater migrations (Groot *et al.*, 1995; Hinch *et al.*, 2005), early marine migrations (Utne *et al.*, 2021) and first marine overwintering period (Duguid *et al.*, 2021). To reach the marine environment, many populations and species of juvenile salmon must first make long, energetically intensive freshwater migrations, during which they may deplete energetic stores as evident by evaluations of stomach fullness, diet composition, energy density and physiological condition (Groot *et al.*, 1995; Hinch *et al.*, 2005; Wilson *et al.*, 2022). Before transitioning to a fully marine environment, juvenile Chinook salmon can reside in estuarine systems, like the Strait of Georgia, for up to several weeks where they can feed to rapidly accrue energy stores and grow (Levy and Northcote, 1982; Moore *et al.*, 2016; Chalifour *et al.*, 2021; Scott *et al.*, 2024). However, juvenile salmon may then become food deprived and suffer from reduced energy stores as they make their early marine migration from these nearshore environments into the fully marine environment, during warm summer months (Utne *et al.*, 2021). Specifically, many juvenile salmon from the Fraser River, like Chinook salmon, may experience food deprivation during their early marine migration as they pass through regions, which have limited prey availability like the Johnstone Strait and Discovery Islands (McKinnell *et al.*, 2014; James, 2019; James *et al.*, 2020; Wilson *et al.*, 2022). In these areas and more widely across the eastern Pacific coast, summer temperatures are warming (Locarnini *et al.*, 2002; Gentemann *et al.*, 2017; Overland *et al.*, 2018; Walsh and Brettschneider, 2019), likely due to the effects of changes in climate (Ministry of Environment, 2016). Further, during early marine residency and migration, in British Columbia (BC), the compounding impacts of temperature stress, pathogens from open net pen salmon aquaculture, and limited food supply often converge and may contribute to the high levels of observed mortality for juvenile Pacific salmon during early marine residency and migration (Brett, 1952; Moss *et al.*, 2005; Duffy and Beauchamp, 2011; Woodson *et al.*, 2013; Vollset *et al.*, 2020; Bateman *et al.*, 2022). Finally, prior to entering the open ocean, Chinook salmon must survive overwintering, a period of low temperatures as well as reduced productivity, which may result in food deprivation, mortality and regulate salmon abundance (Beamish and Mahnken, 2001; Trudel *et al.*, 2012; Hertz *et al.*, 2016). However, at the present time, there is little ability to detect food deprivation and energetic status non-lethally in juvenile Pacific salmon, with current approaches only providing a snapshot of information at the time of capture that can be ambiguous to long-term impacts. Thus, establishing non-lethal food deprivation biomarkers, and incorporating them into a larger sampling framework, can provide a more comprehensive view of food deprivation and the compounding stressors, which are contributing to the declines of wild Pacific salmon.

Lab-based experiments suggest that Pacific salmon exhibit a strong capacity to persist in periods of food deprivation across an array of environmental temperatures (Bilton and Robins, 1973; Snyder, 1980; Ferguson *et al.*, 2010; Seong *et al.*, 2012; Stormer and Juanes, 2013; Wilson *et al.*, 2021; Wilson *et al.*, 2022). These studies demonstrate that juvenile Pacific salmon can survive extended periods of food deprivation, but with impacts to body mass, energy content and physiological condition (Bilton and Robins, 1973; Snyder, 1980; Ferguson *et al.*, 2010; Seong *et al.*, 2012; Wilson *et al.*, 2021; Wilson *et al.*, 2022). Currently, in wild salmon, energetic status is often assessed by length and weight metrics or plasma insulin-like growth factor-1 (IGF-1) levels predictive of instantaneous growth (Beckman, 2011; Ferriss *et al.*, 2014), but these metrics are not always a strong measurement of long-term growth or the impacts of food deprivation (Yakar *et al.*, 1999; Allard and Duan, 2018). In laboratory experiments with Chinook salmon, food deprivation results in the modification of pathways involving protein metabolism, protein degradation and glucose metabolism in the muscle and liver (Esmaeili *et al.*, 2021; Esmaeili *et al.*, 2023). These observed changes in muscle and liver pathways induced by food deprivation suggest that there may be more widespread alterations to transcriptional processes that could be further characterized in metabolically active tissues like the liver and contrasted to more peripheral tissues like the gill, which could then be readily sampled non-lethally to assess energetic status of wild salmon. Overall, these starvation and food limitation experiments provide context for the physiological responses and survival capabilities of Pacific salmon across life stages, and suggest that juvenile Chinook salmon may be able to resist starvation for periods of 30 to 60 days even under elevated temperatures (Snyder, 1980; Stormer and Juanes, 2013). However, these studies do not provide tools that can be readily applied to non-lethally assess the impacts of food deprivation on wild populations. Instead, developing tools that can be used to non-lethally sample wild fish can allow for the sampling of species and populations of concern, and promote the integration of tissue sampling into tagging projects, so that the assessment of food deprivation can be related to the movement and survival outcomes for individual salmon (Thorstensen *et al.*, 2022).

With the advent of novel sampling and molecular approaches (Jeffries *et al.*, 2021; Thorstensen *et al.*, 2022), researchers can now study impacts at the cellular level, which reflect systemic stress. These responses are particularly impactful in the gill, an organ with diverse biological function that serves as an interface between internal and external environments (Gilmour and Perry, 2018) and one that can be readily non-lethally sampled. This rapid responsiveness makes the gill particularly useful for non-lethal sampling in contrast to much less reactive peripheral tissues such as fin clips. Messenger RNA (mRNA), extracted from the gill, has been shown to work in conjunction with high-throughput qPCR platforms to rapidly assess the presence and abundance of pathogenic agents as well as the expression of biomarkers that are predictive of specific stressor and disease states (Miller *et al.*, 2016; Akbarzadeh *et al.*, 2018; Houde *et al.*, 2019; Akbarzadeh *et al.*, 2020). The ‘Salmon Fit-Chip’ employs biomarkers panels that run on a high-throughput Fluidigm qPCR platform to enable non-lethal detection of a variety of stressor states from gill tissue, including thermal stress, hypoxic stress and salinity stress, along with pathogens, to evaluate cumulative stressor impacts across Pacific salmon species (Miller *et al.*, 2016; Akbarzadeh *et al.*, 2018; Houde *et al.*, 2019; Akbarzadeh *et al.*, 2020; Bass *et al.*, 2022; Deeg *et al.*, 2022; Teffer *et al.*, 2022; Wang *et al.*, 2023). However, there is a lack of curated mRNA biomarkers for energetic status, a key physiological metric, which all other transcriptional and cellular responses are reliant upon (Alberts, 2002; Liu *et al.*, 2006; Somero, 2020). Development of mRNA biomarker assays for detecting food deprivation, and their incorporation into the Salmon Fit-Chip, would not only provide a key measure, adding the capacity to directly evaluate energetic status of individual Chinook salmon during key early life bottlenecks to survival, but also provide enhanced context for the other stressor and disease responses already measured.

In this study, we used physiological measurements and RNA-sequencing (RNA-seq) to evaluate the impacts of food deprivation and identify biomarkers that can be used to non-lethally assess the status of food deprivation in wild Chinook salmon. Using an RNA-seq approach, we aimed to investigate gene-expression responses to food deprivation in the gill, a tissue that can be readily non-lethally sampled, in contrast to responses in the more metabolically active liver. We conducted experimental trials at both cool winter and warmer summer temperatures, conditions under which juvenile Chinook salmon may experience food deprivation (8 and 16°C, respectively). We hypothesized that food deprivation would have a profound impact on the physiology and gene expression profiles of both gill and liver tissues of juvenile Chinook salmon, and that environmental temperature would impact these metrics (Schulte, 2015). We predicted that both the rate of physiological decline and transcriptional responses would be more elevated under warmer summer temperatures than at cooler winter temperatures due to increases in metabolic rate and energy consumption (Schulte, 2015). Finally, we predicted that the analysis of the gill transcriptome would reveal conserved responses to food deprivation, evident across both temperature regimes (8 and 16°C), possibly involved in protein metabolism, nutrient cycling and immune capacity, which could be readily exploited to detect food deprivation. Overall, this study provides an in-depth analysis of the physiological and transcriptional responses of Pacific salmon to food deprivation and provides a strong molecular tool, which can be used to non-lethally assess this critical energy-supply-regulating factor in wild salmon.

## Materials and Methods

This experiment was approved by the Fisheries and Oceans Canada (DFO) Pacific Region Animal Care Committee (AUP 2023–004), which abides by the Canadian Council of Animal Care Standards.

### Study species

Age-0 ocean-type Chinook salmon from the Big Qualicum River Hatchery (Qualicum Beach, BC) were transported to the Pacific Biological Station (Nanaimo, BC) for this study. Full methodological details can be found in Supplementary Methods 2.1.

### Experimental setup and acclimation

Fish were transferred into twelve circular experimental tanks (400 L capacity), placing 50 juvenile Chinook salmon in each (600 total fish). Tank treatments were then quasi-randomly assigned during transfer so that there were triplicate tanks for each of combination of temperature (16°C, warm or 8°C, cool) and feeding (fed or unfed) treatments as follows: 16°C fed, 16°C unfed, 8°C fed and 8°C unfed (3 tanks·treatment^−1^; 150 fish·treatment^−1^). Care was taken during transfer so that fish were evenly distributed across experimental tanks (10 per tank per transfer, rotating through experimental tanks, until all fish had been transferred; quasi-randomly assigned) to minimize any possible stocking-order effects on the experiment. During the transfer process, fish were sedated in communal tanks with MS-222 (dosage 5 mg·L^−1^) and then further anaesthetised during transfer to experimental tanks in buckets (dosage 50 mg·L^−1^) to measure fork length (±0.1 cm) and whole-body mass (±0.01 g). Full methodological details can be found in Supplementary Methods 2.2, and details of the experimental setup can be found in Supplementary Fig. S1A.

### Food deprivation and refeeding

Following acclimation, the food deprivation portion of the experiment began with three possible predetermined trial endpoints to be applied at the treatment level, established through consultation with DFO veterinarians and animal care staff:

1. 10% total mortality in a treatment,
2. A relative reduction of Fulton’s condition factor (*K*) (Fulton, 1911) of 20% in unfed fish when compared to their fed control counterparts in a treatment, calculated as:
  $$ K=\frac{\mathrm{m}\mathrm{ass}\ \left(\mathrm{g}\right)}{\mathrm{total}\ \mathrm{length}\ \left(\mathrm{c}{\mathrm{m}}^3\right)}\ast 100 $$
3. 56 days of food deprivation

whichever came first. Once food deprivation began, all food was withheld from the unfed treatments of both 8 and 16°C while the fed treatments were provided with food at a rate of 0.8% and 2.4% body mass.day^−1^ (as determined by veterinary recommendation for 8 and 16°C, respectively).

Once the predetermined endpoint for the food deprivation portion of the experiment was reached for each treatment, the refeeding portion of the experiment began. To gradually allow gut enzymes to return to full production and avoid negative physiological responses, such as bloating, fish were slowly refed. Refeeding started at a 10% daily ration (based on fed treatment feeding rates of 0.8 and 2.4% body mass.day^−1^ for 8 and 16°C, respectively) and increased +10% daily so that a 100% ration was reached by day 10 of the refeeding processes. Once a 100% food ration was reached, this feeding rate was maintained until the end of the experiment.

### Sampling procedures

At the beginning of food deprivation, every 14 days (at most) during food deprivation, every 7 days nearing termination of the food-deprivation periods, and every 3 days during refeeding, we lethally collected samples of gill, liver, muscle and plasma from each treatment to evaluate the physiological condition of the juvenile Chinook salmon and to collect tissues for transcriptional biomarker development and testing (gill and liver). Prior to sampling, food was withheld for 24 hours to reduce potential measurement variability that could be introduced by inter-individual differences in metabolic state based on food consumption (Clark *et al.*, 2013; Hvas *et al.*, 2020). Full methodological details can be found in Supplementary Methods 2.4 and details for full sampling schedule can be found in Supplementary Fig. S1B.

### Energy density measurement

Muscle energy densities from the final food deprivation timepoint were assessed by first drying the samples in an oven at 80°C similar to the methods used by Johnson *et al.* (2017) and Glover *et al.* (2010), followed by bomb calorimetry using a semi-micro oxygen combustion vessel (1109A). Full methodological details can be found in Supplementary Methods 2.5.

### IGF-1 measurement

Plasma IGF-1 concentrations were measured at the final food deprivation timepoint using a time-resolved fluoroimmunoassay (TR-FIA) developed by Small and Peterson (2005) as modified by Ferriss *et al.* (2014), employing a dissociation-enhanced lanthanide fluorescence immunoassay (DELFIA®, Perkin Elmer) methodology. Full methodological details can be found in Supplementary Methods 2.6.

### RNA extraction and sequencing

In preparation for sequencing, RNA was extracted from gill and liver tissues, checked for quality and integrity, and finally shipped to Canada’s Michael Smith Genome Sciences Centre (Vancouver, BC, Canada). Homogenization of gill and liver tissue and total RNA extraction were performed following previously established methods (Houde *et al.*, 2019) with some modifications. From the final food deprivation timepoint, 15 gill and liver samples were selected from each temperature and feeding treatment (*n* = 120 samples total, 60 per tissue) for sequencing based on their RNA quality. Both gill and liver tissues were chosen for sequencing to transcriptionally validate the anticipated metabolic compromise of the liver demonstrating energetic compromise at the transcriptional level following food deprivation in contrast to the less metabolically active gill tissue. Full methodological details can be found in Supplementary Methods 2.7.

### Transcriptome alignments

Following sequencing, raw reads were checked for quality, aligned to the Chinook salmon genome, and then assembled into transcripts. Raw reads were joined and checked for quality using FastQC version v0.12.1 (Andrews, 2010). Using STAR (Dobin *et al.*, 2013; Dobin and Gingeras, 2015), paired read libraries were then aligned to the Chinook salmon, Otsh_v2.0 genome (Christensen *et al.*, 2018) and StingTie version 2.2.1 (Kim *et al.*, 2015; Pertea *et al.*, 2015) was used to produce a set of assembled transcripts for each sample. STAR was run with default parameters and produced an overall alignment rate of approximately 90.2% (± 2.2% SD) and 92.1% (± 0.7% SD) for each gill and liver sample, respectively. STAR-produced binary alignment and map files were then input into StringTie to assemble transcripts, using default settings, and then merged within each tissue type to produce a master list containing 47 063 transcripts in the gill and 35 196 transcripts in the liver. Full details of read mapping for each individual sample can be found in Supplementary File S1.

### Differential expression and functional enrichment

To assess transcriptional differences between fed and food-deprived individuals, bioinformatic approaches were used to identify differentially expressed transcripts and evaluate the processes that these transcripts are involved in for both gill and liver tissue. Full methodological details can be found in Supplementary Methods 2.9.

### Biomarker identification

To identify highly differentially expressed transcripts to be used as candidate biomarkers for food deprivation in the gill (the target tissue of our existing set of nonlethal “Fit-Chip” biomarker assays) and ensure biomarkers could be readily detected during high throughput-qPCR application, conserved transcripts were initially filtered for a baseline expression of >2 counts per million (CPM). Next, the log_2_ CPM values of gill transcripts that were conserved across temperature treatments and were differentially expressed at log_2_FC > 2 were assessed to determine whether transcripts were upregulated or downregulated. As there were no conserved biomarker candidates that were strongly upregulated (log_2_FC > 2) in food-deprived individuals, the analysis was conducted again, lowering the log_2_FC threshold to >1 (twice as highly expressed) to identify consistently upregulated biomarker candidates (though at a lower threshold than we originally anticipated). The inclusion of upregulated biomarkers is critical as they can provide enhanced detection power with increased accuracy and specificity (Su *et al.*, 2019) and are broadly applicable across seasons. One additional criterion—low inter-individual variability, identified as coefficient of variation <50%—was applied to identify biomarkers, which exhibited limited variability in their expression under fed conditions (candidate biomarkers and their CPM values can be found in Supplementary File S5). Overall, this approach resulted in a final set of 19 transcriptional biomarkers for testing. Full methodological details can be found in Supplementary Methods 2.10

### Biomarker assay development, testing and application

TaqMan assays were developed targeting each of the 19 candidate biomarkers discovered in gill tissue through the biomarker identification process (Table 1). Full methodological details can be found in Supplementary Methods 2.11.

**Table 1:** TaqMan assays and PCR efficiencies of candidate biomarkers across eight salmonid species

Symbol	Chromosomes	Primers and Taqman probes	PRC efficiency (%)							
			*O. tshawytsha*	*O. kisutch*	*O. keta*	*O. grobuscha*	*O. nerka*	*S. salar*	*S. trutta*	*S. alpinus*
			(Chinook)	(Coho)	(Chum)	(Pink)	(Sockeye)	(Atlantic)	(Brown)	(Char)
col1a_2_v1	16	F- GACCAAAGGGAGCCAATGGT	143.1	123.6	107.7	104.9	107.6	132.7	118	108.1
		R- CCAGACAGACCTATCAGACCTTGA								
		P-CCTGGCAAGGCAG								
										
col1a_2_v2	7	F-CCTAAGGGAGACAGAGGTGACAA	105.2	103.9	160.9	122.3	111.6	101.2	107.9	102.9
		R-TTGAACCGTCTTTGCCAGAAG								
		P-AGAAGGGACCTGAGGGT								
										
col9a1a	2	F-GCTGGGCCCGATGGA	105.7	115.4	130.8	124.5	110	172.7	124.3	101.4
		R-TCACCTGGACTGCCAACTTTC								
		P-ATTGCCAGCATCTAA								
										
col9a2	13	F-TGGTTCTGAAGATGCTGCAAGA	101.5	102.1	131.1	114.7	117	121.6	103.6	141.8
		R-ACCCAGTACGGCACGCTTAG								
		P-CGGCTGTAGCGGTGAG								
										
col9a3	22	F-ATGGCAGGAGCTGATGGTTT	93.7	93.2	166.5	105.6	118.6	99.4	88.1	120.4
		R-GACTCTCCTTTTTGGCCAACTG								
		P-AGAATGGAGAACCTGGTCC								

(Continued)

**Table 1:** Continued

Symbol	Chromosomes	Primers and Taqman probes	PRC efficiency (%)							
			*O. tshawytsha*	*O. kisutch*	*O. keta*	*O. grobuscha*	*O. nerka*	*S. salar*	*S. trutta*	*S. alpinus*
col10a1a	8	F-CCAAAGGGATATAAGGGAGATCAG	116.6	102.5	154.6	138.7	123	112.2	100.2	120.5
		R-GCACCAGTTGGCCCTGTT								
		P-CAGGGCAAGCAAGG								
										
gatad2a	11	F-GTTTCAATCGCACAGAGGTCCTA	122.3	109.9	131.5	124.3	122.1	**-**	**-**	**-**
		R-GCATTGTTAAATATCAGCTTAGCGTCTA								
		P-TCAGTAAAGGTTCGGTTGTG								
										
nt5dc1	5	F-TTGCAGATCTCCCTCTGGACTAC	106.7	133.6	112.9	100.2	117.9	74.3	113.2	80.3
		R-CCGGTGGTGCAGGGTTT								
		P-CCAGGTTCTCTCCC								
										
mfap2	7	F-CACTGTGCACTGACCTCAACAA	97	110.7	100.6	101.5	98.3	103.9	101.6	100.9
		R-CAGAGGCAGTGGGATGATGAT								
		P-AGGCATTATCAGCACTC								
										
cenpp	7	F-AGTGGAGTTTGAGCTCACTGATGT	104.1	101.3	176.2	119.1	115.4	106.3	96.1	117
		R-AACTCAGTGCTGTCCATGACGAT								
		P-ACGATCACTGACCTCAA								

(Continued)

**Table 1:** Continued

Symbol	Chromosomes	Primers and Taqman probes	PRC efficiency (%)							
			*O. tshawytsha*	*O. kisutch*	*O. keta*	*O. grobuscha*	*O. nerka*	*S. salar*	*S. trutta*	*S. alpinus*
cbln4	7	F-GAGAATGCAGACAGACCAAAGGT	104.8	103.1	172.4	180.7	149.9	**-**	**-**	**-**
		R-TGTTGGTGATGACCCTTCTGTATAC								
		P-CCTTCTCTGCTGGTTTG								
										
bglap	27	F-TGGCCATCCTTGTCCTCTGT	97.3	89.5	95.7	123.7	121.5	103.9	104.7	**-**
		R-GCTTTGAGGCAGTGGAGGTAGA								
		P-TGTGTGTGCCACTCTA								
										
klf15	7	F-CAGTGTCCAGTGTGTGAGAAGAAA	124.3	120.9	149.6	185.1	111.4	118.6	98.1	117.7
		R-TGGACTTTGATGTGTTTTGACAGAT								
		P-TTGCCCGCAGTGAC								
										
egr1	8	F-CCTGATATCTCCATGAACTGTGAGA	125.9	131.8	127.7	121.5	125.5	110.3	134.4	141.1
		R-TGAAGCGGCCAGTGTAGGA								
		P-CAGCGGCTGCCTC								
										
frrs1	10	F-CCATATTTGTACTGTATACCCCCATGT	104.6	112.1	140.3	142.3	120.6	111.3	110	121.4
		R-TAAAACGACTTCCTCTGTCACAAATAA								
		P-TCCCCTACATGTAACTAA								

(Continued)

**Table 1:** Continued

Symbol	Chromosomes	Primers and Taqman probes	PRC efficiency (%)							
			*O. tshawytsha*	*O. kisutch*	*O. keta*	*O. grobuscha*	*O. nerka*	*S. salar*	*S. trutta*	*S. alpinus*
acad11	12	F-AGCGCTCTCTCTGGTCTGAGA	95.4	99.6	119.7	97.1	106.3	105.3	106.7	99
		R-ATATAAATGCGTGCCAAACTGTATG								
		P-TTCCTGCAGGCCGC								
										
spryd3	2	F-TGCCAGCAGGAGGGTTCTAC	125.2	97.8	147.1	110.8	107.1	82	142.8	165.4
		R-GCAGGTCAACCCTGATTTTCTC								
		P-CTACCATCGGCATGATG								
										
hmcn1	3	F-TTCCCTCGATGAACCCCTTA	109.4	124.5	135.7	132.1	114.6	119.7	116.4	174.6
		R-GGTCCCTATGTTGATGGAGCTTT								
		P-CTGTGGGCAGTAATG								
										
cyp2j2	1	F-TGATCTGCTGTCTTGTGTTTGGT	118.4	110.7	157	142.7	128.6	112.2	119.6	126.9
		R-GGAGGGTTTGGAATTGGTCAT								
		P-CCGCTTTGAATACAGC								

### Data exploration and classification

A random forest classification model was trained using normalized qPCR results for two thirds of the sampling data set for the 18 candidate biomarkers that passed quality control (the assay for *spryd3* did not pass quality control for all samples during application), across salmon from both 8 and 16°C treatment and control groups, from the end points of all trials. Samples chosen for RNA-sequencing were excluded from both classification development and testing and were pooled across temperatures for each of the fed and unfed treatments (*n* = 76 training, *n* = 38 testing). Classifier training was done using 10 000 tree iterations (Toth *et al.*, 2019). The random forest model was used to rank the biomarkers and select the best ones for the classifier as described by Akbarzadeh *et al.* (2024) by assessing the performance of the classification on the remaining one third of the testing dataset (*n* = 19 pooled across temperatures for each of the fed and unfed treatments; *n* = 38). The strongest 12 biomarkers were then further assessed and run on the total 560 gill samples that passed quality control from the time course of the experiment. Full methodological details can be found in Supplementary Methods 2.12.

### ANOVA-based statistical analysis

Body morphometrics and physiological data collected at the final food deprivation timepoint (condition factor, HSI, energy density and IGF-1 concentration) were analyzed using two-factor ANOVA including temperature, treatment and its interactions in the model as fixed effects. Physiological data collected throughout the full time series of food deprivation and refeeding (length, mass, condition factor and HSI) were analyzed using two separate three-factor ANOVA, one focusing on the food deprivation portion of the experiment, with one focusing on the refeeding portion of the experiment. To analyze the expression of candidate biomarkers at the end of food deprivation, two-factor ANOVA were used including temperature, treatment and its interactions in the model as fixed effects. Next, biomarker-specific three-factor ANOVA were run to analyze the expression of the strongest 12 biomarkers. These biomarkers-specific three-factor ANOVA, and those for physiological data, included temperature, treatment, timepoint and their interactions in the model as fixed effects. For all biomarker models, data were retained in its untransformed CPM or log_2_ value. Finally, some fish in fed treatments exhibited an unfed classification signature following random forest classification, which we refer to as potential false detections. To investigate if there was evidence of physiological impacts that may provide further context for these detections, these potential false detections, we used a one-factor ANOVA to investigate differences in condition factor and hepatosomatic index (HSI) between potential false detection fish and fed and unfed fish at the end of food deprivation timepoint. Following the evaluation of main and interactive effects, post hoc tests were performed with Tukey’s HSD test from the multcomp package for all ANOVA (Hothorn *et al.*, 2008). Full results of all ANOVA analysis can be found in Supplementary Table S2. All statistical analyses were performed using R 4.4.2 (http://www.R-project.org/) with a significance level (α) of 0.05.

## Results

### Physiological responses to food deprivation

**Figure 1:**
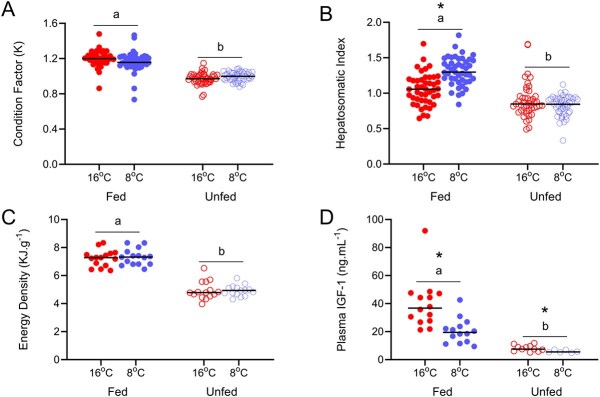
**A**) Condition factor (K), **B**) HSI, **C**) energy density and **D**) plasma IGF-1 concentration at the end of food deprivation in fed versus unfed juvenile Chinook salmon (*O. tshawytscha*) acclimated to 8 and 16°C. Asterisks represent a significant difference between acclimation treatments while lowercase letters represent a significant difference between fed and unfed treatments (*n* = 6–45).

Both fed and unfed juvenile Chinook salmon in 8°C suffered no mortality throughout the 56-day experimental period. However, unfed salmon reared at 16°C reached 10% mortality by day 35, ending the high-temperature portion of the food deprivation experiment. During the period of food deprivation, unfed Chinook salmon did not grow in length and in this warm temperature and had declined in mass, relative to starting conditions, by day 28 (*P* < 0.05; Supplementary Fig. S2A and B). Further, at the end of the food deprivation portion of the experiment there were substantial decreases in condition, HSI, muscle energy density and plasma IGF-1 for both temperature treatments (*P* < 0.05; Fig. 1).

In unfed fish, condition factor (K) declined in comparison to their fed control counterparts by 14 days of food deprivation in both 8 and 16°C (Supplementary Fig. S2C) and had decreased by 19.1 and 16.4% by the end of the food deprivation, respectively (*P* < 0.05; Fig. 1A). Similarly, HSI began to drop (Supplementary Fig. S2D) after 28 days of food deprivation in cool temperatures and 14 days in warm temperatures and decreased by 17.1 and 58.9%, respectively, at the end of food deprivation (*P* < 0.05; Fig. 1B). Muscle energy density decreased consistently in both cool and warm temperatures, with declines of 48.6 and 46.4%, respectively, between fed and unfed treatments (*P* < 0.05; Fig. 1C). Finally, plasma IGF-1 levels were depressed in unfed treatments at the end of food deprivation, 71.2 and 79.6% lower than in fed control fish in cool and warm temperatures, respectively (*P* < 0.05; Fig. 1D).

**Figure 2:**
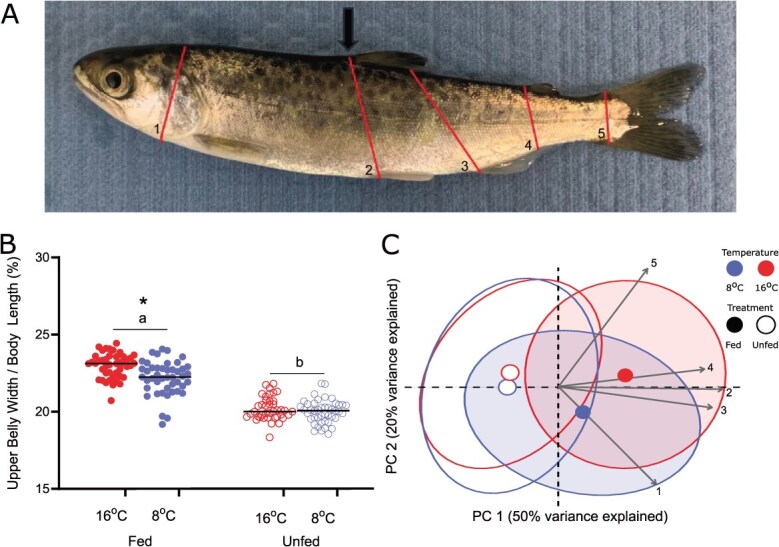
**A**) Image of a juvenile Chinook salmon (*O. tshawytscha*) showing five morphometric measurements highlighted in red (from left to right, 1) head, 2) upper belly, 3) lower belly, 4) trunk and 5) tail; see Supplementary Table S1 for full details on morphometric measurement landmarks and Supplementary Fig. S3 for further body metric ratios measurements) with an arrow indicating **B**) upper belly width body metric ratio and a **C**) PCA and loadings of the total variation based on these five body metric ratios at the end of food deprivation between fed versus unfed fish acclimated to 8 and 16°C. In Panel B, asterisks represent a significant difference between acclimation treatments while lowercase letters represent a significant difference between fed and unfed treatments (*n* = 40–45). In Panel **C**, ellipses represent 95% confidence intervals while points represent the centroid for each treatment group (*n* = 40–45).

Body metric ratios (Fig. 2A) demonstrated slimming, relative to fed control counterparts, of the fish by the end of food deprivation, across different body proportions in both 8 and 16°C. By the end of the trial, unfed fish from both cool and warm temperature treatments had upper belly width to length ratios that were decreased by 12–13% across temperature treatments, (*P* < 0.05; Fig. 2B) and a reduction between 3 and 10% of the other body metric ratios, when compared to fed control counterparts (*P* < 0.05; Supplementary Fig. S3A–D). However, when incorporating all five body metric ratios into a principal component analysis (PCA), there was some evidence of separation between fed and unfed treatments along PC1 (50% variance) but no clear separation from either feeding treatment or temperature based on visual markers (Fig. 2C). Overall, the body metric ratios representing belly and trunk width were co-aligned and drove variation along PC1, while variation along PC2 was mostly driven by divergent trajectories for head and tail width.

During the refeeding portion of the experiment, unfed fish began to recover rapidly, resuming growth in 16°C and an increase in condition and HSI during this recovery period in both 8 and 16°C (Supplementary Fig. S4). In warm temperatures, food-deprived fish demonstrated increases in mass beginning at day 12 and length at day 21 of refeeding (*P* < 0.05; Supplementary Fig. S4A and B). Similarly condition and HSI rapidly rebounded in unfed fish following refeeding. Condition increased in the unfed treatment beginning at day 12 of refeeding in warm temperatures and returned to the same relative condition of previously fed control counterparts by day 12 in both temperature treatments (*P* < 0.05; Supplementary Fig. S4C). In both temperature treatments, HSI recovered in unfed fish and rapidly increased past the level of previously fed control fish by days 9 and 6, respectively. Unfed fish far surpassed the HSI of their fed control counterparts peaking at a 92% increase at days 18 of refeeding in cool temperatures and a 227–255% increase at days 9–12 in warm temperatures, relative to the previously fed control fish at the same time points (*P* < 0.05; Supplementary Fig. S4D).

### Transcriptional responses to food deprivation

**Figure 3:**
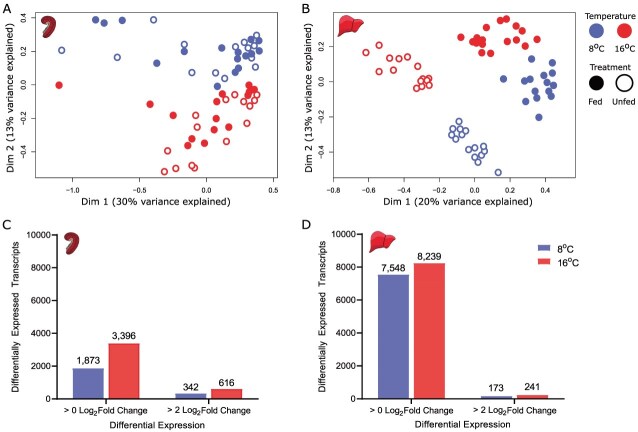
Multidimensional scaling analysis of transcripts in the **A**) gill and **B**) liver as well as overall differential expressed transcripts in the **C**) gill and **D**) liver of fed versus food-deprived juvenile Chinook salmon (*O. tshawytscha*) at 8 and 16°C as measured using RNA-sequencing. Differentially expressed transcripts are represented by an absolute value log_2_-fold change >0 (representing all significantly differentially expressed transcripts) and highly differentially expressed transcripts with an absolute log_2_ fold change >2. Transcript numbers represent either a significant upregulation or downregulation as detected with a *q* value (false discovery rate adjusted *P*-value) < 0.05.

Multidimensional scaling analysis of all transcripts from the gill and liver reveal divergent responsive patterns between the two tissues (Fig. 3). While the expression of transcripts in the gill demonstrated some separation between temperatures of 8 and 16°C along dimension 2 (13% variance), there was little to no separation between fed and food-deprived individuals in the multidimensional scaling analysis (Fig. 3A). In contrast, in the liver both temperature and feeding treatments group out separately along dimension 1 (20% variance) and dimension 2 (13% variance; Fig. 3B).

Similarly, patterns of overall differential expression differ between the gill and liver. In the gill 5269 total transcripts were differentially expressed (>0 log_2_FC) while 958 of these transcripts were highly differentially expressed (>2 log_2_FC; Fig. 3C). In contrast, in the liver there were approximately three-fold as many total differentially expressed transcripts with 15 787, but with less than half as many highly differentially expressed transcripts with 414 (Fig. 3D).

Across both tissues the vast majority of these highly differentially expressed transcripts were unique to each temperature treatment, leaving only a small portion conserved across temperatures as candidate biomarkers. Of these highly differentially expressed transcripts (>2 log_2_FC) in the gill, there were 283 unique to 8°C, 557 were uniquely differentially expressed at 16°C and 59 were conserved between the two temperatures. In the liver, 90 transcripts were unique to 8°C, 158 were uniquely differentially expressed at 16°C and 83 were conserved between the two temperatures.

The majority of all functional enrichment terms were categorized as gene ontology (GO) biological processes, encompassing 65% of all GO terms identified and highlighting changes in molecular processes, which contribute to a larger biological objective (Thomas, 2017), and therefore, these terms were focused on for further analysis to evaluate overarching pathways, which were altered by food deprivation.

**Figure 4:**
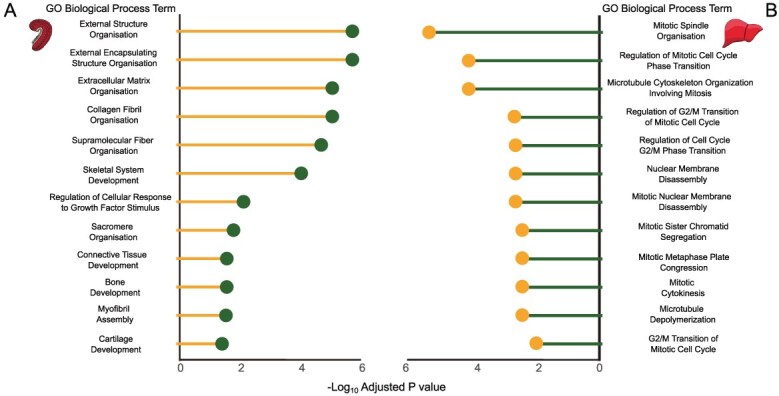
GO biological process terms from highly differentially expressed transcripts (>2 log_2_FC) conserved in both 8 and 16°C in the **A**) gill and **B**) liver of fed versus food-deprived juvenile Chinook salmon (*O. tshawytscha*) as measured using RNA-sequencing. Terms represent all 12 GO biological processes in the gill, and the top 12 GO biological processes in the liver. All terms presented are statistically significant at a false discovery rate adjusted *P*-value <0.05.

In the gill, biological processes modified by food deprivation were involved in structural components and development of the tissue itself (Fig. 4A; Supplementary Fig. S5). That is, processes with the smallest *P*-values were involved in organization of external cellular structures like the extracellular matrix. Further, the development of specific structural components like collagen, cartilage, bone, myofibrils and other connective tissues was downregulated by food deprivation. Finally, there were alterations in responses to growth factors (Supplementary File S3, Sheet A).

In the liver, biological processes modified by food deprivation were involved in cell cycling, fatty acid regulation and processing of secondary metabolites (Fig. 4B; Supplementary Fig. S6). Processes with the smallest *P*-values were involved in cell cycling with specific emphasis on mitosis and the G2/M damage checkpoint (Fig. 4B). Further, fatty-acid-related processes include modifications to triglycerides, lipids, cholesterol, phospholipids, high-density lipoproteins and hormones in both their metabolism and storage (Supplementary Fig. S6). Finally, there were alterations to biological processes involved with other metabolites including glycerol, glutamate, glycogen, sterol and hydrogen peroxide (Supplementary Fig. S6; Supplementary File S3, Sheet B).

### Biomarker identification, training and testing

**Figure 5:**
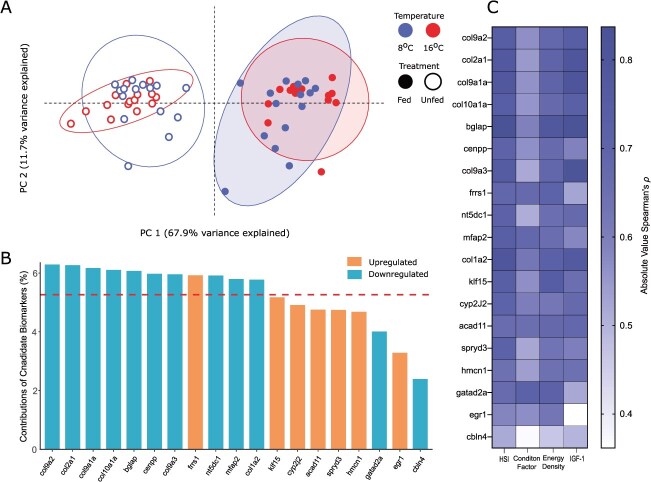
**A**) PCA, **B**) contributions to the variance in the overall PCA and **C**) correlation with physiological metrics (hepatosomatic index—HSI, condition factor, energy density KJ·g^−1^ and IGF-1 ng·ml^−1^) of candidate food deprivation biomarkers conserved in both 8 and 16°C in the gill of juvenile Chinook salmon (*O. tshawytscha*) as measured using RNA-sequencing. In panel A, ellipses indicate 95% confidence regions, while in panel, B the dotted line indicates the expected default contribution of a single candidate biomarker to the overall variation observed in PC1 and PC2.

There were 12 unique gill biomarker candidates, which were consistently downregulated at a log_2_FC > 2 and 7 unique candidates, which were consistently upregulated at a log_2_FC > 1 conserved across both 8 and 16°C temperatures in food-deprived individuals when compared to their fed control counterparts. Biomarker candidates, which were downregulated in food-deprived individuals were associated with collagen formation (*col1a2, col9a1a, col9a2, col9a3, col10a1a, col2a1*), neuronal development (*cbln4, gatad2a*), microfibril formation (*mfap2*) and bone remodelling (*bglap*) consistent with overarching functional analysis (Supplementary Fig. S7). Upregulated biomarker candidates exhibited more diverse functions and were associated with cellular differentiation *(klf15, egr1*), iron metabolism (*frrs1*), fatty acid oxidation (*acad11*), cytoskeletal organization (*spryd3*), immunoglobulin/connective tissue formation (*hmcn1*) and inflammation mediation (*cyp2j2*; Supplementary Fig. S8). A PCA including the CPM expression of all 19 candidate gill biomarkers demonstrated clear separation between fed and unfed treatments, with little impact of temperature (Fig. 5A). The biomarker candidates with the strongest contributions to the overall variation in the PCA were largely made up of the alpha collagen as well as *bglap* and *frrs1* (Fig. 5B). These same biomarker candidates also exhibited the strongest relationship with physiological metrics HSI, condition factor, energy density and IGF-1 (*P* < 0.05; Fig. 5C).

**Figure 6:**
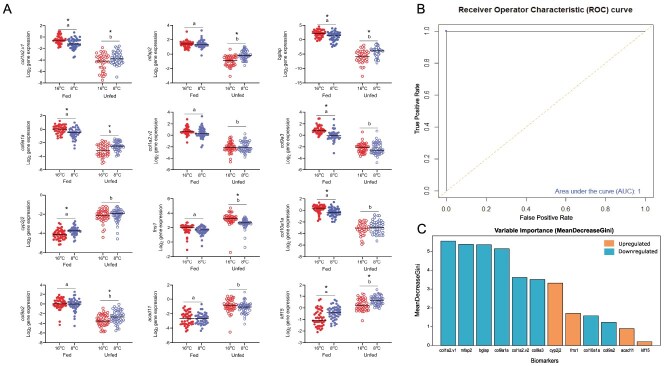
**A**) High-throughput qPCR for 12 biomarkers included in the food deprivation classifier for all samples from the final food deprivation time point (sequencing, testing and training, *n* = 15 each; total *N* = 45) as well as **B**) receiver operator characteristic curve and **C**) Gini scores (a measure of variable importance) for biomarkers from random forest model training. In panel A, asterisks represent a significant difference between acclimation treatments while lowercase letters represent a significant difference between fed and unfed treatments.

**Figure 7:**
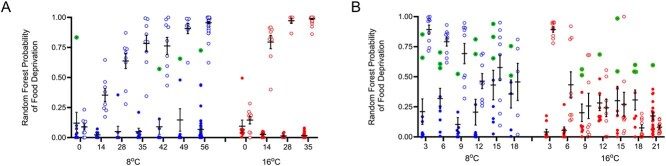
Random forest probability of food deprivation during both **A**) food deprivation and **B**) refeeding in fed versus food-deprived juvenile Chinook salmon (*O. tshawytscha*) at both 8 and 16°C based on classification by the expression of 12 gill transcriptional biomarkers. Fish, which were fed during the food deprivation portion of the experiment, are indicated by solid dots while fish undergoing either food deprivation or refeeding are indicated by hollow dots with temperatures indicated by 8°C in blue (left) and 16°C in red (right) for each figure. Individual points for potential false detections of unfed fish in fed treatments at a random forest classification threshold of 0.5 are distinguished by a green outline (bolded). Horizontal bars indicate the mean of each sample group, while vertical bars and cross represent the standard error of the mean (*n* = 5–45).

The expression of candidate biomarkers was evaluated using high-throughput qPCR assays designed across salmonid species demonstrating strong efficiencies (80–120%) for most primer sets across the 8 salmonid species and their potential for cross-species application (Table 1). Next, using the process of iterative optimization, removing individual biomarkers and retraining the classifier to test its performance, we determined that reducing the total number of biomarkers from an initial set of 18 down to 7 did not impact the classification performance (Supplementary Table S3). Random forest classifiers trained on biomarker panels ranging from 12 (Fig. 6A) to 7 biomarkers showed an area under the receiver operator characteristic curve of 100% (a measure of true positive rate, sensitivity, versus false-positive rate, specificity) on the training set (*n* = 76) demonstrating perfect sensitivity and specificity for classification of both fed and unfed treatments (Fig. 6B). Furthermore, Gini scores (a measure of variable importance) demonstrate that the alpha collagens, *bglap*, *mfap2* and *cyp2j2* made strong contributions to the overall random forest classification (Fig. 6C).

On the testing dataset (*n* = 38), a random forest classification model trained on 12 to 7 biomarkers showed high prediction accuracy of 97.4% and sensitivities of 100 and 94.7% for unfed and fed control fish, respectively. However, when the classifier was reduced to 6 biomarkers, removing the final upregulated biomarker, classifier sensitivity dropped to 84% in fed control fish and prediction accuracy dropped to 94.7%, highlighting the importance of including upregulated biomarkers for classifier training and testing (Supplementary Table S3). Full results can be found in Supplementary Results 3.3.

#### Biomarker detections throughout food deprivation and refeeding

No biomarkers were differentially expressed at either 8 or 16°C at the beginning of trials, but all biomarkers became differentially expressed by 14 days at 16°C and 28 days at 8°C (*P* < 0.05; Supplementary Fig. S9). In cool temperatures, biomarkers became differentially expressed either by 14 days (*bglap, col9a1a, col1a2.v2, col9a3, cyp2j2, acad11* and *klf15*) or by 28 days (*col1a2.v1, mfap2, frrs1, col10a1a* and *col9a2*; Supplementary Fig. S9A–L). Once differentially expressed, these differences in biomarker expression between fed and unfed fish persisted until the end of food deprivation and into refeeding. Full results can be found in Supplementary Results 3.4.

### Random Forest classification throughout food deprivation and refeeding

The random forest-based model applied both during food deprivation and refeeding demonstrated strong classification success throughout the entirety of the experiment at a random forest classification threshold of 0.5 (Fig. 7).

At the beginning of food deprivation trials, 97% of all fish were classified as fed with the consistent classification of unfed fish beginning at day 14 in 16°C and day 28 in 8°C (Fig. 7A). By day 14 of food deprivation 52.3% of unfed fish across the study were classified as food deprived (12.5% in 8°C and 88.9% in 16°C). Further, by day 28 of food deprivation, the percentage of food-deprived fish continued to increase with 86.7% of unfed fish classified as food deprived (77.8% in 8°C and 100% in 16°C). At day 35, the final day of food deprivation for warm temperature exposed juvenile Chinook, 98.1% of all sampled fish were classified as food deprived (88.9% in 8°C, 100% in 16°C). Finally, with only cool temperature exposed salmon remaining for days 42, 49 and 56 of the food deprivation experiment, 89, 100 and 100% of fish were classified as food deprived, respectively. Pooling Chinook from across both temperatures and time points throughout the entirety of the food deprivation period, the mean random forest probability of food deprivation for fish in the fed treatment was 5.5%, while it was 83.4% from days 14 onward in the food deprivation treatment (Fig. 7A).

Throughout the refeeding period the food deprivation signature waned from 100% following 3 days of refeeding to 21 and 0% at the final refeeding timepoints, for 8 and 16°C, respectively (Fig. 7B). By 6 days of refeeding this proportion dropped to 66.7% (100% in 8°C, 33.3% in 16°C). The percentage of fish classifying as food deprived continued to drop on days 9 and 12 to 47 and 31%, respectively. Finally on days 15, 18 and 21 of refeeding, the percentage of fish classifying as food deprived dropped to 29, 21.4 and 0%, respectively (55% in 8°C and 3% in 16°C, throughout this time period). While many of the unfed fish had returned to fed status by day 12 of refeeding, 8.1% of the remaining fish sampled from day 12 to 21 in warm temperatures and 50% of those sampled in cool temperatures had persistent transcriptional signatures of food deprivation demonstrating inter-individual variation in the return to fed status and the potential for longer-term impacts of food deprivation.

Throughout food deprivation and refeeding, there were a total of 26 potential false detections of food deprivation in the fed treatment (Fig. 7; individuals outlined in green). However, examining physiological status of all potential false detections classified as food deprived at a probability threshold greater than 50% revealed (*n* = 26) that these individuals had lower condition and HSI than their fed control counterparts at the end of food deprivation (*P* < 0.05; Supplementary Fig. S13A and B) suggesting a real physiological impact in conjunction with the transcriptional responses highlighted by random forest classification and not true false detections. Furthermore, at the later portion of this refeeding period (days 12–21), an increase in food deprivation signature to an intermediate status representing a random forest classification probability of approximately 15–25% was observed in both fed and unfed treatments. The probability results of the random forest classification analysis for all 559 sampled individuals are available in Supplementary File S7.

## Discussion

Juvenile Chinook salmon were exposed to extended periods of food deprivation, followed by refeeding, which induced strong impacts on their physiology and transcriptome. RNA-sequencing revealed gill mRNA biomarkers that can be used non-lethally to assess food deprivation and recovery. Physiologically, there were decreases in overall condition, HSI, plasma IGF-1 and muscle energy density, which were associated with suspended growth in food-deprived fish, while transcriptionally, there were impacts on the metabolic processes of the liver, and structural components of the gill. By evaluating these impacts across both cool winter and warm summer (8 and 16°C), we identified an optimized and flexible panel of differentially expressed gill biomarkers representing key structural components and physiological processes affected by food deprivation. Using the expression of genes in this panel, in conjunction with random forest classification, revealed strong overall classification performance throughout food deprivation and refeeding as well as clear temporal patterns that could be assessed with high accuracy and precision. With the assays used in this study designed to perform across salmonid species, this biomarker panel provides a novel molecular tool that can be readily applied to non-lethally assess food deprivation in wild Chinook salmon and other salmonids.

### Physiological responses to food deprivation

Extended periods of food deprivation had strong impacts on the physiology of juvenile Chinook salmon held at both warm summer and cool winter temperatures, with stark decreases in body metric ratios, condition factor, HSI, muscle energy density and plasma IGF-1 levels at the end of food deprivation. Decreases in condition and HSI in food-deprived salmon appeared at 14 days in 16°C and 28 days in 8°C. Together, these metrics can be indicative of fatty acid and glycogen stores accrued during development (Chellappa *et al.*, 1995; Purchase, 2001; Bugg *et al.*, 2020; Morrison *et al.*, 2020). Thus, their more rapid decline in warm temperatures suggests earlier constraints on metabolic budgets induced by food deprivation, likely as a result of increases in the rates of biological processes such as metabolic rate in elevated temperatures (Tomanek and Somero, 1999; Schulte, 2015; Somero, 2020). Large decreases in plasma IGF-1, a strong indicator of growth rates for Chinook salmon (Duguid *et al.*, 2018), and muscle energy density assessed in this study were similar to decreases observed in sockeye salmon following food deprivation during ocean entry and in laboratory experiments where swimming performance and survival were impacted (Wilson *et al.*, 2021; Wilson *et al.*, 2022; LoScerbo *et al.*, 2024). Further, the above findings are consistent with impaired metabolic rate following food deprivation in Atlantic salmon, *Salmo salar*, smolts (Hvas *et al.*, 2020).

Observed body metric (belly, trunk and tail width) to length ratio decreases during food deprivation were similar to those observed previously in food-deprived chum salmon (Seong *et al.*, 2012). These ratios could provide an accurate method to assess physiological metrics that are difficult to measure rapidly and accurately in field settings (Holmes and Jeffres, 2021). While the strongest body metrics (like upper belly width) provide important easily visible physiological context of food deprivation, there was enough individual variability overall to prevent the clear identification of food-deprived individuals using body metric ratios alone.

The rapid recovery of HSI during refeeding to levels surpassing the fed control group supports the findings of previous food deprivation studies on other teleosts. While HSI recovered to the state of fed control fish within 3 days of refeeding, it greatly surpassed the levels of fed control fish by as much as 250%. Rapid elevation of HSI during refeeding, indicating increases in liver size, also coincided with increases in length and mass. Similar rebounds and elevations of HSI during refeeding have been observed in flounder, *Paralichthys olivaceus*, Atlantic cod, *Gadus morhua* and Nile tilapia, *Orechromis nilticus*, following periods of food deprivation (Black, 1986; Cho, 2005; Nebo *et al.*, 2018), suggesting that this relative increase in HSI is a common response during refeeding. While commonly observed across fish taxa, there are few hypotheses presented to explain its occurrence. In humans, short periods of fasting followed by refeeding can lead to increases in overall liver volume as well as hepatic fat content (Šedivý *et al.*, 2024). Thus it is likely that, in salmon, this relative increase in liver size is a temporary mechanism of energy storage either in the form of glycogen or fatty acid reserves, which were likely depleted during food deprivation, and that these stores can then be subsequently used to fuel growth as fish slowly transition to a recovered status during the refeeding period. Overall, the rapid recovery of HSI and condition and the return of growth for unfed fish suggests that by day 12 of refeeding Chinook salmon begin to recover physiologically, repleting these hepatic energy stores and remobilizing accrued energy for growth.

The combination of the physiological metrics measured here reflects the regulation of growth that can ultimately influence size-selective mortality in wild fish (Beamish and Mahnken, 2001; Moss *et al.*, 2005; Duffy and Beauchamp, 2011; Woodson *et al.*, 2013). In this study, these metrics provide compelling evidence that unfed fish were strongly physiologically impacted by the effects of food deprivation. These measurements, like body morphometrics, which can be readily assessed in the field, can be further paired with transcriptional responses and other physiological data to more holistically develop our understanding of the continuum of changes to overall fish condition that occur throughout food deprivation.

### Transcriptional responses to food deprivation

Transcriptional profiles of the gill and liver revealed distinct tissue-level responses to starvation and, in the gill, biomarkers predictive of food deprivation. Transcriptional shifts in the liver were characterized by genes involved in cell cycling and the shift from carbohydrate metabolism to gluconeogenesis and fatty acid oxidation as energetic reserves dwindled (Qian *et al.*, 2016; Zhu *et al.*, 2020; Chen *et al.*, 2023; Lin *et al.*, 2023; Xie *et al.*, 2023), similar to observations of altered cellular metabolism and turnover in food-deprived chum (Seong *et al.*, 2012). However, in the gill, highly differentially expressed transcripts were associated with collagen fibrils and the extracellular matrix, as well as transcripts associated with the processing of fatty acids and iron. These gill biomarkers were highly correlated with the more traditional physiological metrics that were impacted by food deprivation including HSI, body condition, muscle energy density and plasma IGF-1. Together, as the gill biomarkers identified are composed of highly conserved structural components (Newstead, 1967; Bettex-Galland and Hughes, 1973), as well as metabolic processes induced by food deprivation (Nabatame *et al.*, 2021; Paquay *et al.*, 2024), it is likely that they will be effective in detecting food deprivation across salmonids and perhaps in other fishes as well.

In the gill, the downregulation of collagen alpha chains, *bglap* and *mfap2* during food deprivation represents the compromise of both structural and functional components of the gill lamella in food-deprived Chinook. In fact, the strongest pattern induced by food deprivation was the consistent downregulation of a variety of collagen alpha chains, which together form the structural basis of the lamellae of teleost gills (Newstead, 1967; Garnero, 2015). In addition to the downregulation of collagen itself, *bglap*, a gene encoding for the protein osteocalcin, which functions as the main producer of type 1 collagen by crystallizing the collagen network (Wei and Karsenty, 2015), was also heavily depressed in unfed fish. In *bglap* knockout mice, the collagen crystallization process was disrupted, leaving behind disordered mineralization and reduced structural integrity of the collagen framework (Moriishi *et al.*, 2020). In fish, this loss of gill structural integrity may be critical, as the collagen scaffolding supports a network of pillar cells, which regulate blood flow to the respiratory surfaces of the gill through contraction, in turn regulating oxygen uptake (Newstead, 1967). These pillar cells rely on *mfap2*-produced elastin microfibrils to contract (Weinbaum *et al.*, 2008; Craft *et al.*, 2010; Craft *et al.*, 2012), reducing the lamellar space and increasing functional surface area promoting gas exchange between the vasculature of the gill and the external environment (Newstead, 1967; Bettex-Galland and Hughes, 1973; Laurent and Dunel, 1980; Hughes, 1984; Nilsson and Sundin, 1998). Together the downregulation of these transcripts demonstrate that the structure, integrity and respiratory function of the gill are impaired by food deprivation.

In contrast, the upregulation of *klf15*, *acad11*, *cyp2j2* and *frrs1* represents gill mechanisms that were induced to produce energy and make structural components more bioavailable during food deprivation. *Klf15* is a transcription factor, which regulates the metabolic switch between the consumption of glucose and fatty acids (Nabatame *et al.*, 2021; Shochat *et al.*, 2021). As metabolism shifts towards fatty acid consumption, *acad11* promotes the entry of long chain fatty acids into the peroxisome and mitochondria (He *et al.*, 2011; Paquay *et al.*, 2024) where they go through beta-oxidation to produce energy. These long chain fatty acids are typically beta oxidized under severe starvation scenarios, after available glucose reserves and shorter chain fatty acids have been metabolized, as this process can result in the production of reactive oxygen species, which can be damaging to the cell (Jezierska *et al.*, 1982; Soeters *et al.*, 2012; Silva-Marrero *et al.*, 2017; Zengin, 2021). However, another upregulated biomarker, *cyp2j2*, functions to produce epoxyeicosatrienoic acids, which act as signalling molecules to prevent the formation of reactive oxygen species and to promote anti-inflammatory responses (Thomson *et al.*, 2012; Shochat *et al.*, 2021). The final upregulated biomarker *frrs1* converts ferrous iron to ferric iron, making it more bioavailable to act in multiple biological processes, including as a key post-translational modifier and structural component of collagen (O’Dell, 1981). Overall, the combined upregulation of these genes during food deprivation suggests that the complex of *klf15*, *acad11* and *cyp2j2* are co-regulating the switch to long chain fatty acid metabolism as energetic reserves become depleted, while *frrs1* is making iron more bioavailable, likely to strengthen existing structures during an overall loss of lamellar integrity.

The food deprivation classifier based on the above biomarkers demonstrated high accuracy with random forest classification probabilities of 97.1% for unfed fish and 4.2% for fed control fish and a potential false-positive rate of 1.1% (a single false detection) at the end of food deprivation. The classifier was sensitive enough to begin detecting food deprivation as early as 14 to 28 days in 16 and 8°C, respectively, with detections persisting at least 6 days following refeeding. Importantly, the sensitivity to detect these earlier impacts of food deprivation suggests that the classifier has the ability to accurately assess the levels of food deprivation that may be expected for wild juvenile salmon both during early migrations and the beginning of overwintering as well as the more severe impacts of prolonged periods of starvation (Triebenbach *et al.*, 2009; Duguid *et al.*, 2021; Wilson *et al.*, 2021). Overall, the classifier demonstrates strong performance throughout food deprivation and the ability to distinguish between fed and food-deprived individuals earlier in the refeeding and recovery process than traditional physiological metrics like body condition. Together, these findings suggest that these gill biomarkers paired with high-throughput qPCR and random forest classification produce a powerful tool that is well suited for the non-lethal detection of transcriptional responses representative of food deprivation in the gill of wild salmon.

### Inter-individual variation in transcriptional responses

There was considerable inter-individual variation in the transcriptional signatures of unfed fish returning to fed status during the refeeding process. While most fish returned to fed status fairly rapidly, within 9 days for both temperature treatments, some fish in warm temperatures took up to 15 days, and approximately half in cool temperatures took at least 18 days to return to fed status based on gill transcriptional patterns. This suggests an impact of temperature with unfed salmon in 16°C returning to fed status more rapidly and with less variability than those 8°C, consistent with the generally anticipated metabolic impacts of temperature (Tomanek and Somero, 1999; Schulte, 2015; Somero, 2020). Further, during this refeeding period some fed control fish began to show signs of starvation with more potential false-positive detections of food deprivation in fish from fed treatments. However, upon investigation these individuals with potential false detections demonstrated reduced condition and HSI when compared to fed control counterparts suggesting real intermediate-level detections of reduced physiological performance that may have compromised the physiological status of some fish in fed treatments during the refeeding period. These intermediate detections may be the result of disruptions, changing tank dynamics or the formation of dominance hierarchies (Ejike and Schreck, 1980; Sloman *et al.*, 2000) as the number of individuals in each study tank rapidly decreased due to sampling following the end of food deprivation and throughout the refeeding period.

Overall, the differences in classification probabilities between fed and unfed fish were stark once the impacts of food deprivation were apparent (<10% for fed individuals and >80% for unfed individuals at the end of food deprivation). Further, there was a short period (14 to 28 days) where unfed fish demonstrated intermediate detections both during food deprivation and refeeding (ranging from 30 to 50% classification probability). This result suggests the potential for a continuum of effects, which increase as the impacts of food deprivation become more severe. Overall, the detection of inter-individual variation in the return to fed status, differences in the rate of temperature regulated impacts of food deprivation, intermediate physiological impacts for fish in fed treatments, and the potential for a continuum of detections across fed and unfed states highlight the effectiveness of the food deprivation classifier to reveal food deprivation signatures that may otherwise go undetected.

### Study limitations

Although the findings of this study are consistent throughout the time series of food deprivation and refeeding, and with a broader literature surrounding the impacts of food deprivation, there are some limitations to the inferences that can be drawn from the results. First, the Chinook salmon used in this experiment were sourced from the Big Qualicum River Hatchery and thus have altered exposure to environmental conditions when compared to wild fish, which may result in different physiology and responses to the stressors presented in this experiment. Second, as the fish used in this study were from the same age group, and warm and cold temperatures occur at different times of the year naturally, there is the potential for life-stage-environment mismatch on the physiological responses of the fish observed here. However, as the observed transcriptional and physiological responses are strongly conserved across temperature treatments this is unlikely. Next, the differentially expressed pathways and biomarkers highlighted in this study were detected using RNA-sequencing and evaluated by applying high log fold change thresholds to isolate highly differentially expressed genes. This process may have obscured some of the more subtle physiological processes that could be impacted by food deprivation like amino acid recycling. Further, using RNA-sequencing restricts these molecular characterisations of the impacts of food deprivation to the transcriptional level, where many genes and proteins are pleiotropic and often are involved in numerous functional processes (Caspari, 1952; Watanabe *et al.*, 2019). Finally, individual-level random forest probabilities for the detection of food deprivation during the later portion of the refeeding process are likely impacted by inter-individual interactions and intratank effects, which hamper our interpretation of the food deprivation signature at the end of this period. Despite these limitations, the biomarkers identified and random forest classifier built in this study demonstrate high accuracy and low false-positive rates, demonstrating strong performance and the ready application for the detection of food deprivation in wild fish.

## Conclusions, impacts and future work

Determining the impacts of multiple stressors is critical for predicting how organisms will respond to shifting and compounding stressors; however, the causes of early marine mortality for salmon are clouded by these interactions (Beamish, 2022). The food deprivation biomarkers discovered and developed in this study will be deployed alongside an array of qPCR biomarker panels known as the ‘Salmon Fit-Chip’ enabling the high-throughput, non-lethal detection of a variety of stressor states from gill tissue, including temperature, oxygen, salinity and morbidity, as well as disease states (Dittmar *et al.*, 2014) and infectious agents in wild Pacific salmon (Report on the Performance Evaluation of the Fluidigm BioMark Platform for High-Throughput Microbe Monitoring in Salmon, 2016; Akbarzadeh *et al.*, 2018; Houde *et al.*, 2019; Akbarzadeh *et al.*, 2020). As such, the deployment of these novel food deprivation biomarkers in wild Chinook salmon will not only provide a targeted view of energetic status but will also enhance insights into insights into the roles that these interconnected factors play in determining survival outcomes (Jeffries *et al.*, 2014; Teffer *et al.*, 2022), which ultimately regulate evolutionary trajectories for populations and species (Dittmar *et al.*, 2014; Strowbridge *et al.*, 2021). By pairing food deprivation biomarkers with biotelemetry (Duguid and Juanes, 2017) and conducting widespread sampling across life stages, researchers can provide new insights into how food deprivation contributes to physiological stress and ultimately mortality (Keagy *et al.*, 2023), paving the way for more effective management strategies.

## Supplementary Material

Web_Material_coaf088

## Data Availability

Much of the data used in this study is available in the included Files (Supplementary Files S1–S7) and the binary alignment and map files produced and used in this study are available at the National Centre for Biotechnology Information Sequence Read Archive with the accession number PRJNA1302434. Further, all Supplementary files are publicly available on figshare at: 10.6084/m9.figshare.30696236.
