# Supplementary material for: Identification of food deprivation in salmonids using gill biomarkers
Source: Conserv Physiol. 2025 Dec 19;13(1):coaf088. doi: 10.1093/conphys/coaf088 (PMC12716027; doi:10.1093/conphys/coaf088)
Supplement: Web_Material_coaf088 [file web_material_coaf088.zip › Food Deprivation Figures - Revised - Supplemental.docx]

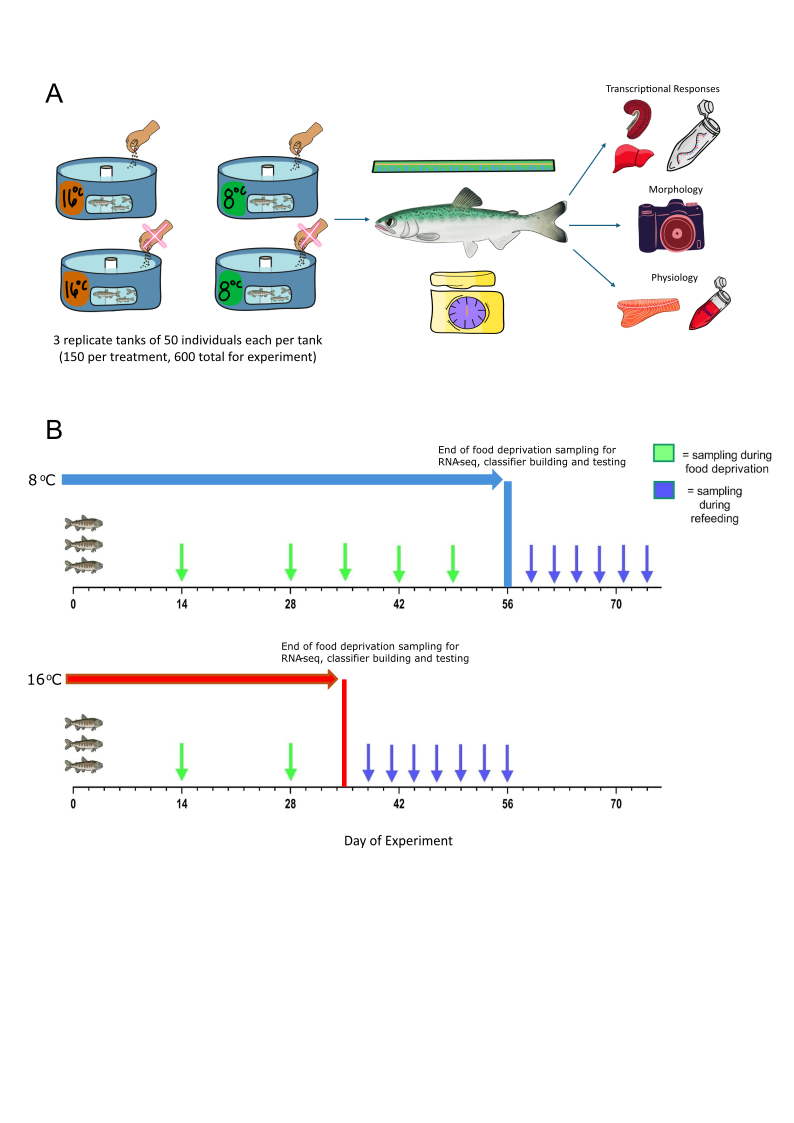


Supplemental Figure 1. A) Experimental design and B) sampling schedule for food deprivation experiments in juvenile Chinook salmon (*Oncorhynchus tshawytscha*) at both 8 and 16^o^C. Illustrations by Madison Earhart. Each blue arrow during the food deprivation portion of the experiment represents the sampling of 9 salmon, while each green arrow during refeeding represents the sampling of 3, aside from the final sampling point for 8^o^C during which all remaining fish were sampled. Vertical blue and red bars indicate the end of the food deprivation portion of the experiment, and the sampling of 45 salmon from each treatment.


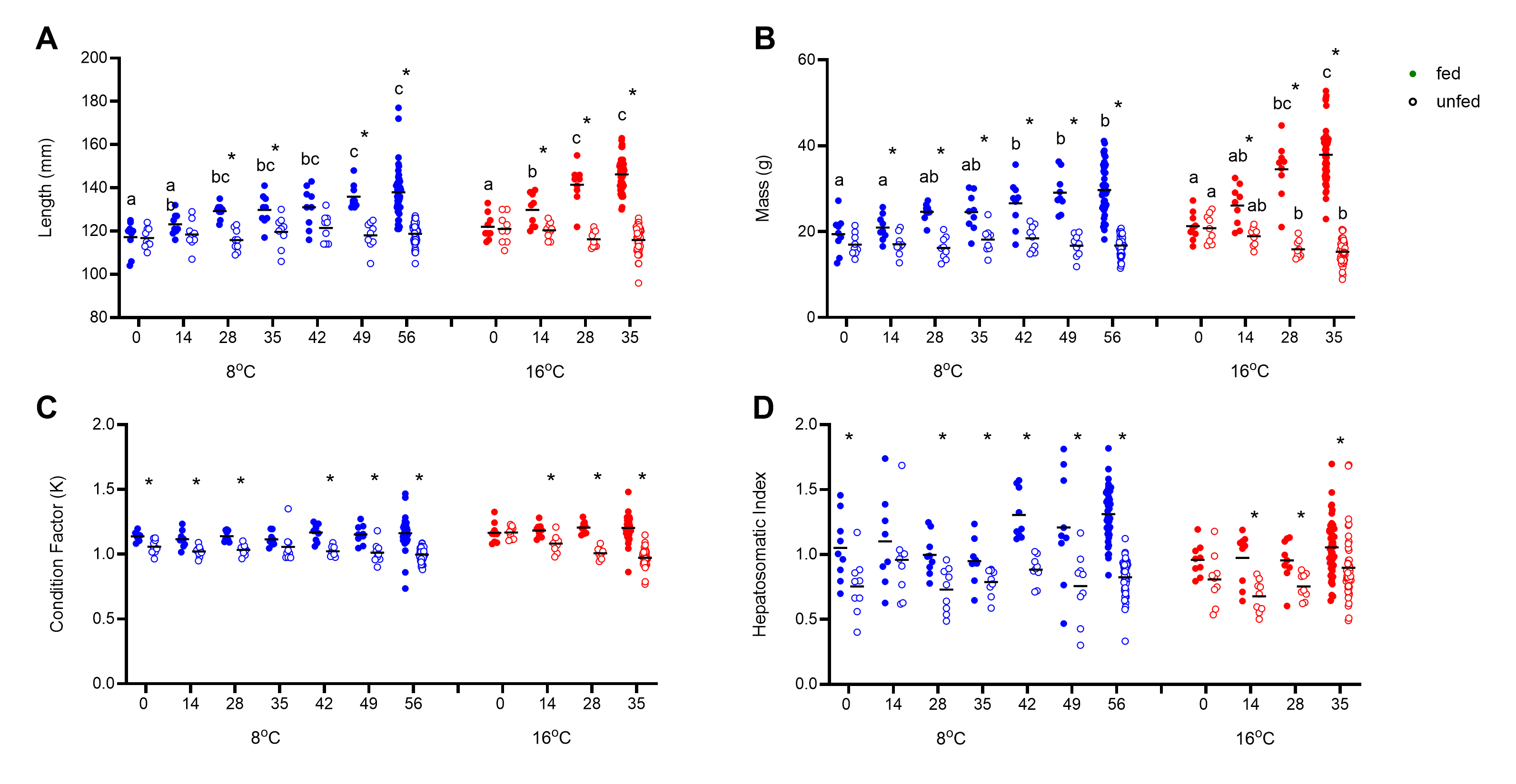


Supplemental Figure 2.  Full food deprivation time series for A) length, B) mass, C) condition factor, D) hepatosomatic index of fed versus unfed juvenile Chinook salmon (*Oncorhynchus tshawytscha*) at both 8 and 16^o^C.  Fish which were fed during the food deprivation portion of the experiment are indicated by solid dots while fish undergoing refeeding are indicated by hollow dots with temperatures indicated by 8^o^C in blue and 16^o^C in red. * indicate a significant difference between fed and unfed fish at given time point and temperature, while different letters indicate significance throughout time, within a temperature and treatment group (P < 0.05; three-factor ANOVA). Solid black bars indicate the mean of each sample group (n = 9-45).



Supplemental Figure 3. Morphometrics at end of trial A) trunk, B) tail, C) lower belly, and D) head width (see Supplemental details on morphometric landmarks) relative to body length (%) at the end of food deprivation in fed versus unfed juvenile Chinook salmon (*Oncorhynchus tshawytscha*) acclimated to 8 and 16^o^C. Fish which were fed during the food deprivation portion of the experiment are indicated by solid dots while fish undergoing refeeding are indicated by hollow dots with temperatures indicated by 8^o^C in blue and 16^o^C in red. Asterisks represent a significant difference between acclimation treatments while lowercase letters represent a significant difference between fed and unfed treatments and solid black bars indicate the mean of each sample group (n = 40 - 45).





Supplemental Figure 4. Full food deprivation refeeding and recovery time series for A) length, B) mass, C) condition factor, D) hepatosomatic index of fed versus food deprived juvenile Chinook salmon (*Oncorhynchus tshawytscha*) at both 8 and 16^o^C.  Fish which were fed during the food deprivation portion of the experiment are indicated by solid dots while fish undergoing refeeding are indicated by hollow dots with temperatures indicated by 8^o^C in blue and 16^o^C in red. * indicate a significant difference between fed and refeeding fish at given time point and temperature, while different letters indicate significance throughout time, within a temperature and treatment group (P < 0.05; three-factor ANOVA). Solid black bars indicate the mean of each sample group (n = 6-24).





Supplemental Figure 5. REVIGO (Reduce + Visualize Gene Ontology) plots of GO (gene ontology) biological process terms from highly differentially expressed transcripts (> 2 log_2_FC) in the gill of fed versus food deprived juvenile Chinook salmon (*Oncorhynchus tshawytscha*) at both 8 and 16^o^C as measured using RNA-sequencing. Bubble color indicates the significance of the adjusted p value after applying a log10 scale (darker color is a more significant term), while bubble size indicates the frequency of a GO term in the GO annotation database (larger bubble is a more common GO term in the database).





Supplemental Figure 6. REVIGO (Reduce + Visualize Gene Ontology) plots of GO (gene ontology) biological process terms from highly differentially expressed transcripts (> 2 log_2_FC) in the liver of fed versus food deprived juvenile Chinook salmon (*Oncorhynchus tshawytscha*) at both 8 and 16^o^C as measured using RNA-sequencing. Bubble color indicates the significance of the adjusted p value after applying a log10 scale (darker color is a more significant term), while bubble size indicates the frequency of a GO term in the GO annotation database (larger bubble is a more common GO term in the database).


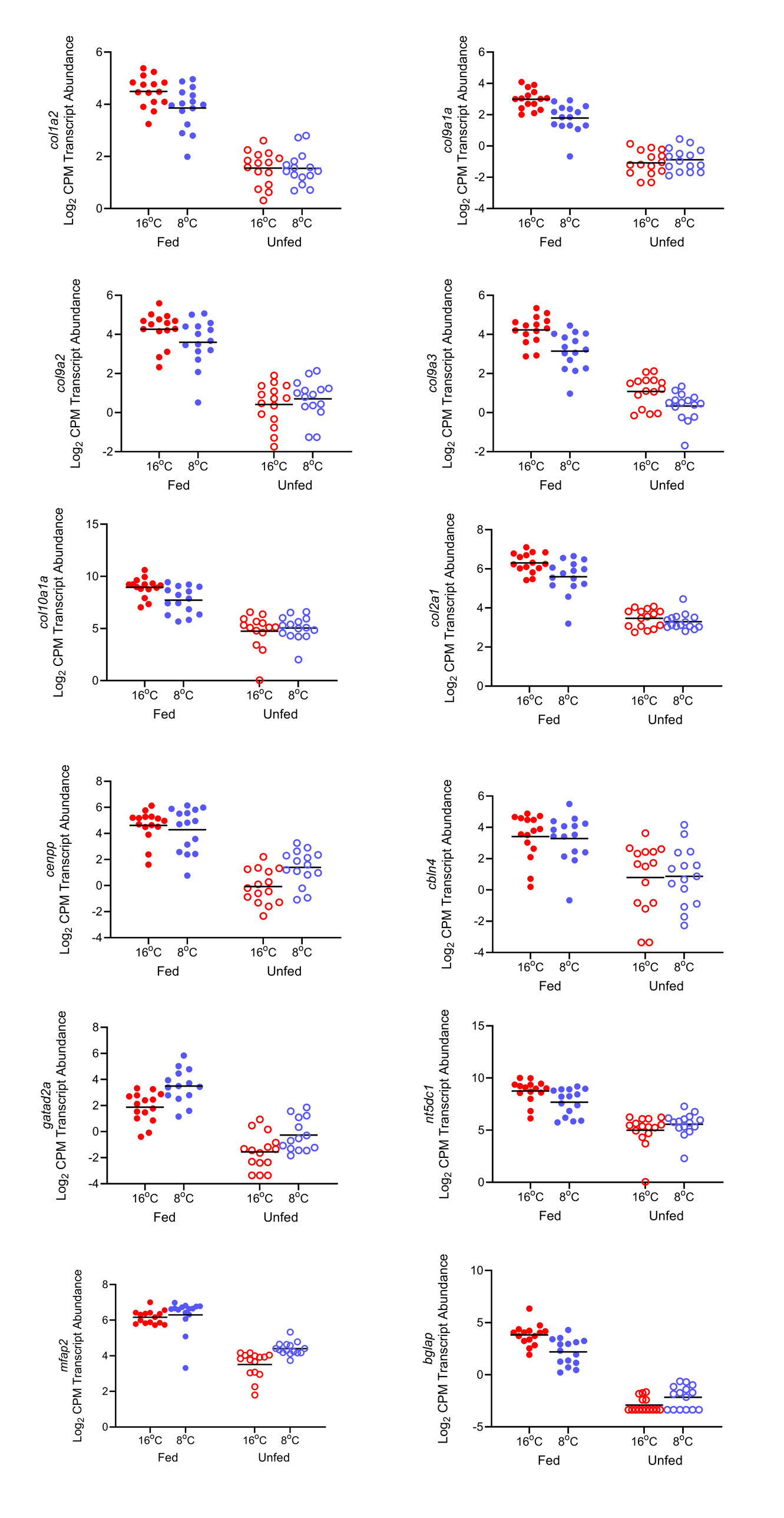


Supplemental Figure 7. Candidate downregulated biomarkers for food deprivation in Chinook salmon (*Oncorhynchus tshawytscha*) which were significantly differentially expressed > 2 log_2_FC between fed and unfed treatments in the gill in both 16 and 8^o^C as measured using RNA-sequencing. Fish which were fed during the food deprivation portion of the experiment are indicated by solid dots while fish undergoing refeeding are indicated by hollow dots with temperatures indicated by 8^o^C in blue and 16^o^C in red. All comparisons between fed and unfed individuals in either temperature treatment are significant P < 0.05.


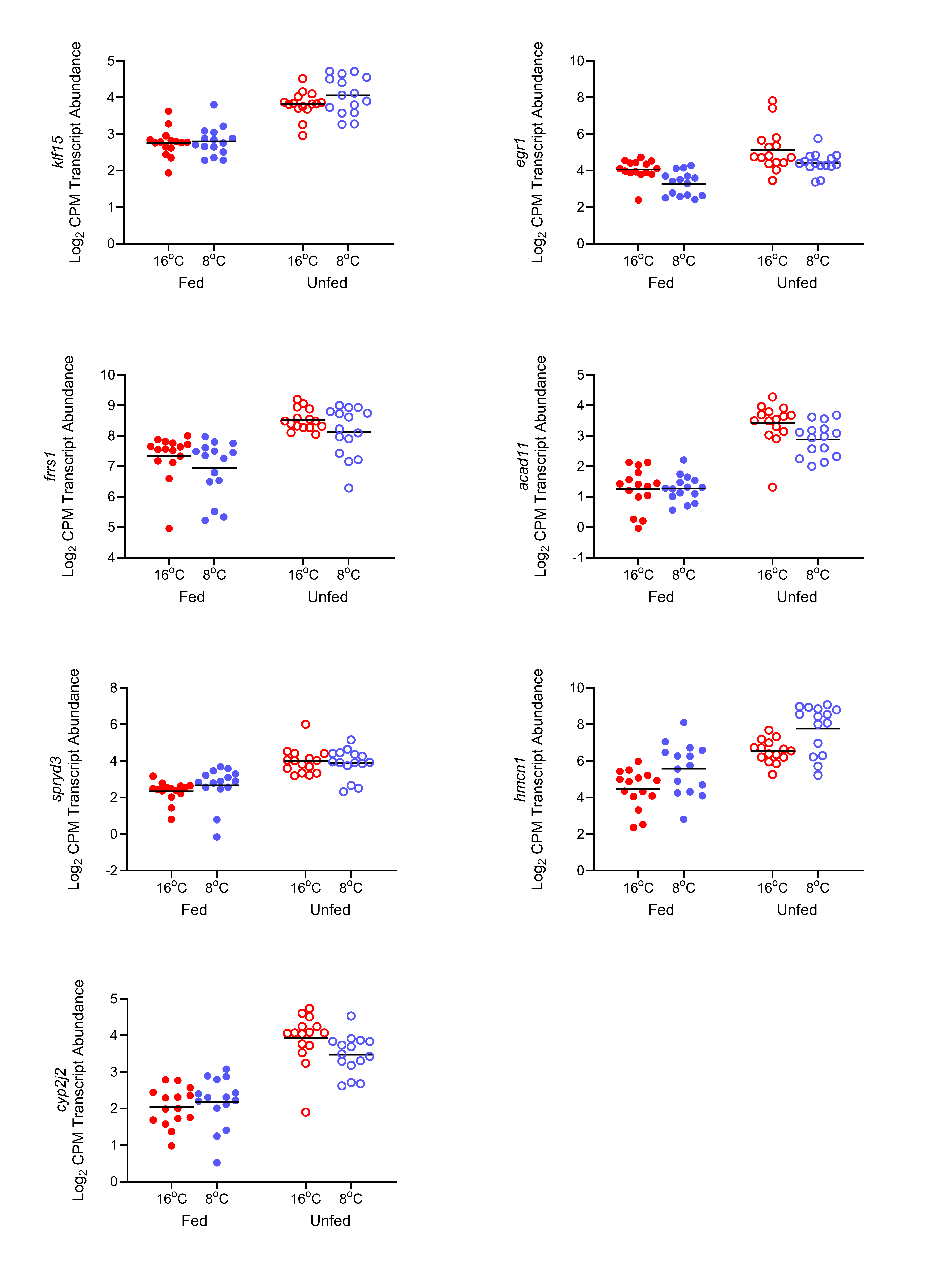


Supplemental Figure 8. Candidate upregulated biomarkers for food deprivation in Chinook salmon (*Oncorhynchus tshawytscha*) which were significantly differentially expressed > 1 log_2_FC between fed and unfed treatments in the gill in both 16 and 8^o^C as measured using RNA-sequencing. Fish which were fed during the food deprivation portion of the experiment are indicated by solid dots while fish undergoing refeeding are indicated by hollow dots with temperatures indicated by 8^o^C in blue and 16^o^C in red. All comparisons between fed and unfed individuals in either temperature treatment are significant P < 0.05.





Supplemental Figure 9. Full food deprivation time series detailing the expression of individual biomarkers A) *col1a2.v1*, B) *mfap2*, C) *bglap*, D) *col9a1a*, E) *col1a2.v2*, F) *col9a3*, G) *cyp2j2*, H) *frrs1*, I) *col10a1a*, J) *col9a2*, K) *acad11*, L) *klf15* in fed versus food deprived juvenile Chinook salmon (*Oncorhynchus tshawytscha*) at both 8 and 16^o^C as measured using high throughput qPCR. Fish which were fed during the food deprivation portion of the experiment are indicated by solid dots while unfed fish are indicated by hollow dots with temperatures indicated by 8^o^C in blue and 16^o^C in red. * indicate a significant difference between fed and unfed fish at a given time point and temperature (P < 0.05; three-factor ANOVA). Solid black bars indicate the mean of each sample group (n = 5-45).





Supplemental Figure 10. Full refeeding time series detailing the expression of individual biomarkers A) *col1a2.v1*, B) *mfap2*, C) *bglap*, D) *col9a1a*, E) *col1a2.v2*, F) *col9a3*, G) *cyp2j2*, H) *frrs1*, I) *col10a1a*, J) *col9a2*, K) *acad11*, L) *klf15* in fed versus food deprived juvenile Chinook salmon (*Oncorhynchus tshawytscha*) at both 8 and 16^o^C as measured using high throughput qPCR. Fish which were fed during the food deprivation portion of the experiment are indicated by solid dots while unfed fish which are now being refed are indicated by hollow dots with temperatures indicated by 8^o^C in blue and 16^o^C in red. * indicate a significant difference between fed and unfed fish at a given time point and temperature (P < 0.05; three-factor ANOVA). Solid black bars indicate the mean of each sample group (n = 5-23).


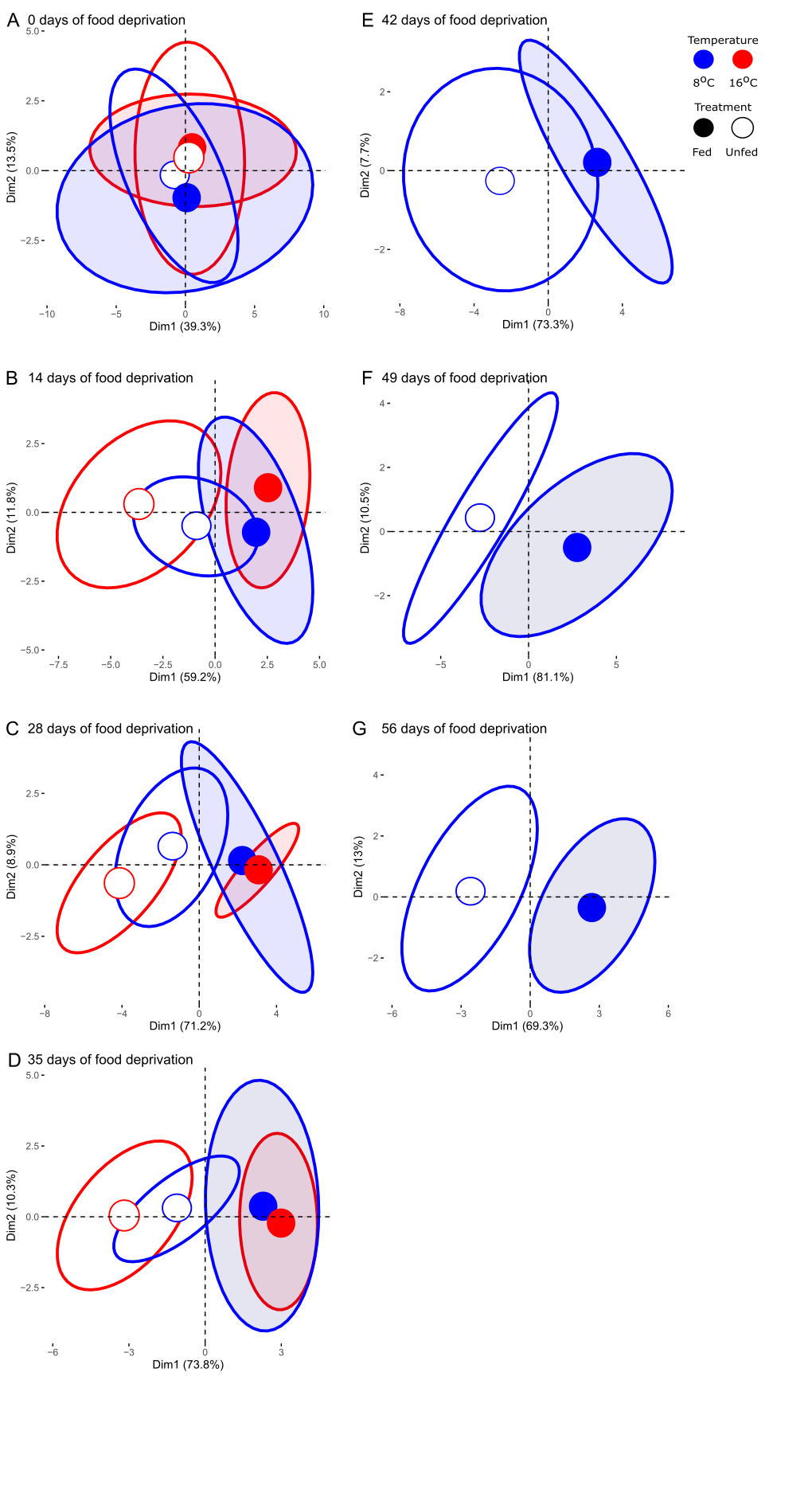


Supplemental Figure 11. Principal component analysis of 12 food deprivation biomarkers in the gill of Chinook salmon (Oncorhynchus tshawytscha) held at 8 and 16oC as measured using high throughput qPCR following A) 0, B) 14, C) 28, D) 35, E) 42, F) 49 and G) 56 days of food deprivation. Ellipses represent 95% confidence intervals while points represent the centroid for each treatment group (n = 6-45).


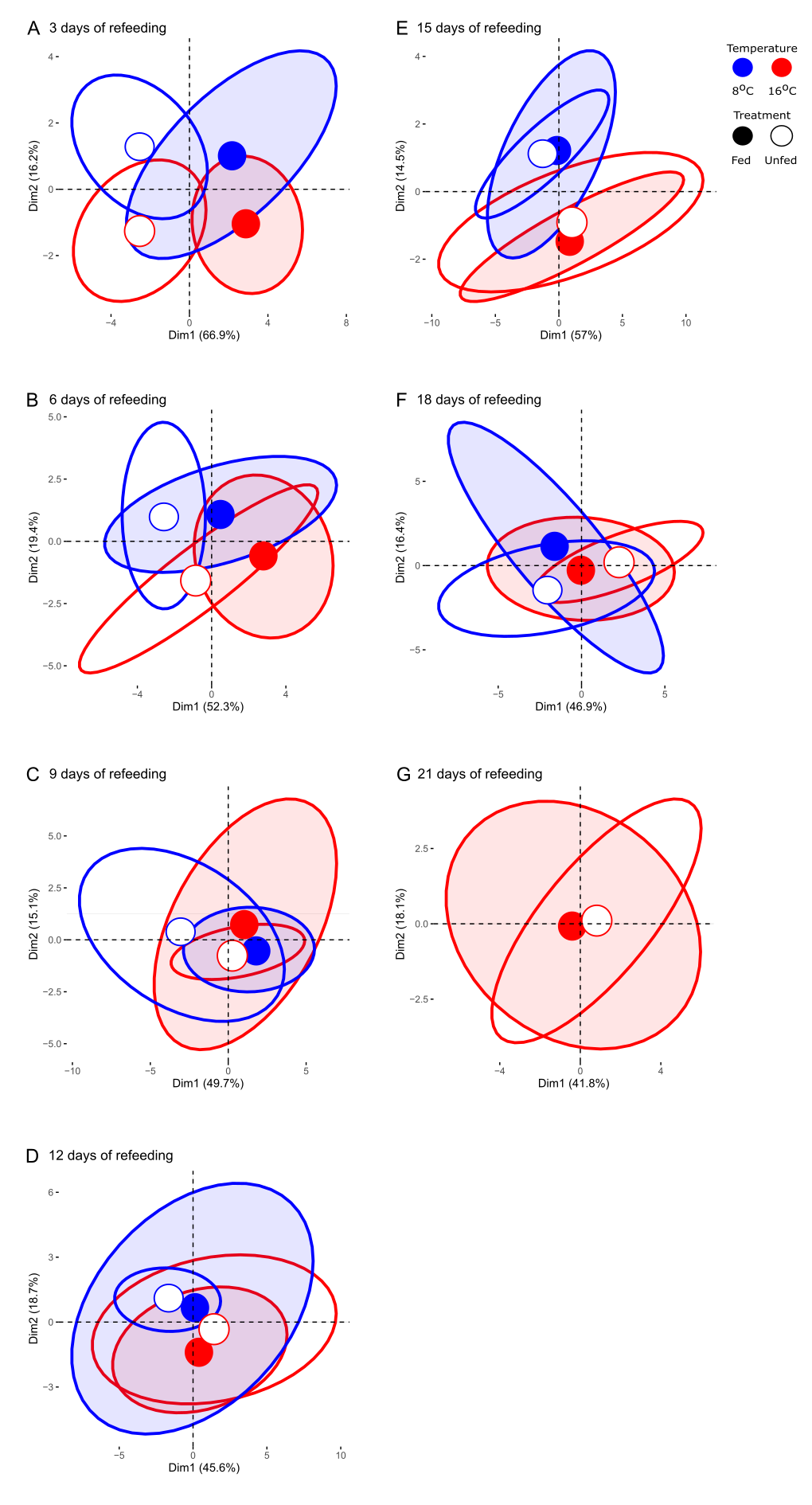


Supplemental Figure 12. Principal component analysis of 12 food deprivation biomarkers in the gill of Chinook salmon (*Oncorhynchus tshawytsch*a) held at 8 and 16^o^C as measured using high throughput qPCR following A) 3, B) 6, C) 9, D) 12, E) 15, F) 18 and G) 21 days of refeeding, after an initial period of food deprivation. Ellipses represent 95% confidence intervals while points represent the centroid for each treatment group (n = 5-23).


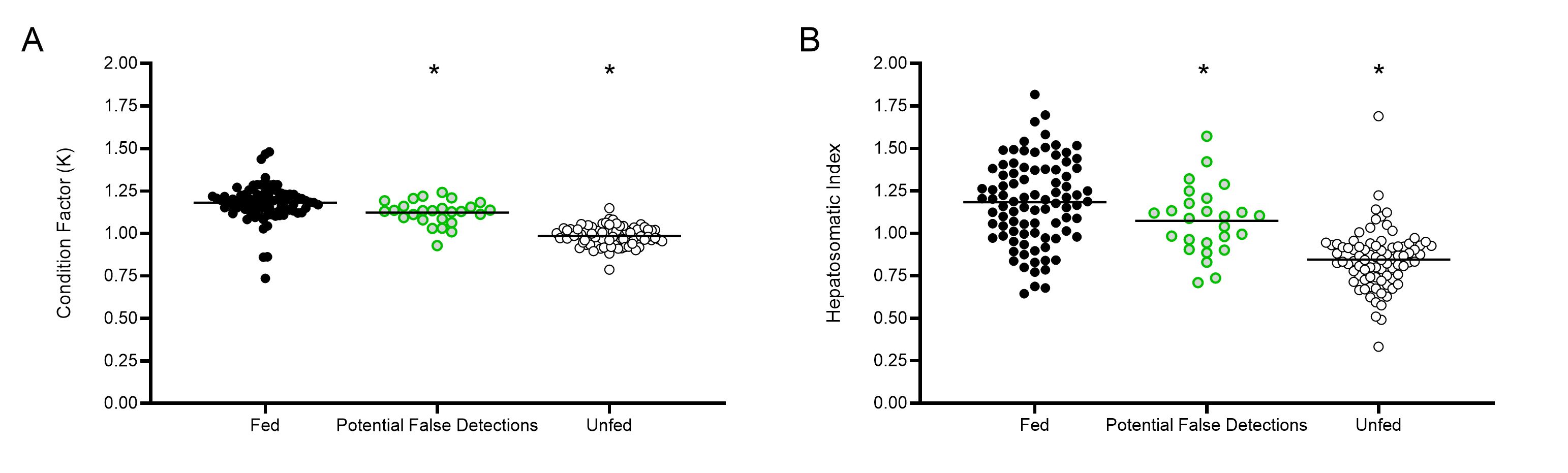


Supplemental Figure 13. A) Condition Factor (K) and B) Hepatosomatic Index of fed, potential false detections, and unfed food deprived juvenile Chinook salmon (*Oncorhynchus tshawytscha*). Fed and unfed fish are salmon sampled from both 8 and 16^o^C at the end of food deprivation, while potential false detections refers to fed fish which were classified as unfed individuals either during food deprivation (4) or refeeding (22). Individual points for potential false detections are distinguished by a green outline matching the physiology of the same individuals in Figure 8. * indicates a significant difference from fed fish (P <0.05; one-factor ANOVA). Solid black bars indicate the mean of each sample group (n = 89, 26, and 84 for fed, potential false detections, and unfed, respectively).
