# Supplementary material for: Identification of food deprivation in salmonids using gill biomarkers
Source: Conserv Physiol. 2025 Dec 19;13(1):coaf088. doi: 10.1093/conphys/coaf088 (PMC12716027; doi:10.1093/conphys/coaf088)
Supplement: Web_Material_coaf088 [file web_material_coaf088.zip › Food Deprivation Supplemental Methods and Results- Revised - Conservation Physiology.docx]

Supplemental Methods

2.1 Study Species

Upon arrival, fish were held in well aerated communal rearing tanks with dechlorinated freshwater at 12°C, under a natural light cycle. Fish were then seawater acclimated for smolting, increasing the salinity 2 ppt per day for two weeks until approximately 28 ppt was reached. This was achieved by supplying UV-disinfected seawater (27–29 ppt salinity) to each tank from Departure Bay, outside the Pacific Biological Station where fish were housed. Post-smolting, juvenile Chinook salmon were allowed to recover in their communal rearing tanks for at least two weeks, until they were transferred to experimental tanks for thermal acclimation and food deprivation trials. Throughout communal rearing and experiments fish were fed Pacific plus complete fish food for salmonids (EWOS, Surrey, BC, Canada).

2.2 Experimental Setup and Acclimation

Following transfer, juvenile Chinook were maintained in the experimental tanks, with tight fitting lids and continuous water flow of 5–7 L.min^-1^ and water velocity of 1–2 body lengths per second for the remainder of the experiment. Incoming seawater was either heated or cooled prior to entering the tanks to reach the desired thermal profile for each treatment. Dissolved oxygen was maintained at 7.5–10 mg.L^-1^ throughout the experiment. Fish were held at 12°C and fed at a rate of 1.2% body mass.day^-1^ for one week until thermal acclimation began.

Thermal acclimation was initiated by increasing or decreasing the temperature of each tank (starting from 12^o^C) at a rate of 2^o^C.day^-1^ until the designated experimental temperatures of 16^o^C and 8°C were reached. Fish were then held at this temperature for one week until food deprivation began. Throughout the course of acclimation and experimentation, observations were conducted at least twice daily to evaluate feeding performance and behavior, record temperature, salinity, and dissolved oxygen levels, and check for any mortality.

2.4 Sampling Procedures

Throughout the food deprivation portion of the experiment, a total of nine juvenile salmon from each treatment (three fish from each of the three replicate tanks.treatment^-1^) were euthanized with an overdose bath of MS-222 (250 mg.L^-1^) made using water of the same temperature and salinity as the experimental conditions for that treatment. At the end of the food deprivation portion of the experiment, 45 juvenile salmon per treatment (15 fish from each of the three replicate tanks.treatment^-1^) were collected, 15 per treatment to be used for RNA sequencing and the remaining 30 per treatment to be split between random forest classifier training and testing.

Following euthanasia, juvenile Chinook salmon were photographed, and measured for fork length (± 0.1 cm), whole-body mass (± 0.01 g), and liver wet mass (± 0.01 g). Using fork length and whole body mass Fulton’s condition factor (K)[^80^](https://paperpile.com/c/Ab0ybX/Jp5SR) was calculated as described above. Further, using liver wet mass and whole-body mass, hepatosomatic index (HSI)[^83^](https://paperpile.com/c/Ab0ybX/B4bmQ) was calculated as: $HSI = \frac{Wliver(g)}{Wbody(g)} * 100$

Finally, using photographs of each individual fish at trial endpoints, changes in body metric ratios of head, upper belly, lower belly, trunk, and tail width (nominators, ± 0.01 cm) to fork length (denominator, ± 0.01 cm) were assessed using standardized physiological landmarks[^84^](https://paperpile.com/c/Ab0ybX/ZUsxe) (see supplemental table 1 for more information). After taking measurements using ImageJ[^85^](https://paperpile.com/c/Ab0ybX/SzJCN), body metric ratios were calculated as:

$$Body Metric Ratio =\frac{Metric Width (cm)}{Fork Length (cm)} * 100$$

Following photographs and initial measurements, tissue samples (blood plasma, gill, muscle, and liver) were collected using a newly sterilized set of tools for each fish. Blood was collected via caudal tail severance using heparinized collection tubes and then immediately centrifuged to separate plasma, which was transferred to a separate eppendorf vial and stored at -80^o^C. Next, dissections for tissue samples were conducted on individually labeled sterilized dissecting mats to avoid contamination among fish. Samples of gill and liver tissue were extracted and then bisected individually, placing one half into a sterile eppendorf vial on dry ice and the other half into *RNAlater* (Invitrogen; Thermo Fisher Scientific). Once sample collection was complete, both sets of gill and liver samples were stored in a -80^o^C freezer until they were used for molecular assessments. Muscle tissue was collected from a standardized sampling location along the side of the fish, starting ventral to the lateral line, just dorsal to the pelvic fin running dorsally to the pectoral fin. After dissection of each fish, dissecting tools were disinfected with an immersion in 10% bleach followed by 95% ethanol and flame, after which the tools were cooled before being used for another fish.

2.5 Energy Density Measurement

Wet mass of each muscle sample was recorded prior to drying. Based on methodology similar to that used by Johnson et al.[^84^](https://paperpile.com/c/Ab0ybX/1m0xz) & Glover et al.[^85^](https://paperpile.com/c/Ab0ybX/6gmwx), samples were dried in an oven at 80°C for a minimum of seven days. Once dry, samples were re-massed and then homogenized by first removing the skin and cutting it into millimeter squares to create uniform pellets. Skin pieces and muscle tissue were both added to a mortar and ground with a pestle until a uniform powder was achieved. Two to three 6.4 mm diameter pellets were made for each fish using a pellet press, depending on tissue size to allow for technical replicates. Pellets were put back in the 80°C oven for at least 2 days to remove any moisture generated during homogenization and pellet formation. Pellets were assayed in an oxygen bomb calorimeter (Parr Company Instruments 6200) using a semi-micro oxygen combustion vessel (1109A). For each sample, at least two pellets were tested separately, with a third pellet tested if the first two results varied by more than 2%. The mean dry energy density value for each sample was converted to energy per wet mass by multiplying by the percent moisture content. Individual moisture percentages were not available for three samples due to scale malfunction, and as such we applied an average moisture percentage for the respective treatment groups, determined by removing the three highest and three lowest moisture values before averaging as is standard for interpolating values.

Prior to running samples in the bomb calorimeter, the effective heat capacity of each oxygen bomb was determined by averaging ten 0.2 g benzoic acid standardization runs, as recommended by the manufacturer. After 10 test sample runs, a standardization test was run to ensure that the resulting energy density did not differ by more than 2% from the known value. The effective heat capacity value for each oxygen bomb remained constant for all test samples.

2.6 IGF-1 Measurement

Ethanal and HCL were added to plasma and vortexted, the resulting supernatants were then assayed in duplicate, alongside recombinant barramundi IGF-1 (GroPep) standards. Polyclonal rabbit anti-recombinant barramundi IGF-1 serum (GroPep) was used as a primary antibody, and tracer was produced by custom labeling recombinant salmon IGF-1 (GroPep) with europium (Perkin Elmer). Reactions were carried out in DELFIA® assay buffer in goat anti-rabbit IGG-coated yellow 96-well plates (Perkin Elmer) and samples were randomly distributed across the plates.

All samples and standards were analyzed in duplicate to assess coefficient of variation (CV). Data was processed using MARS Software (BMG Labtech) with a four-parameter logistic equation to fit the standard curve. Blank corrected absorbance values were used to calculate binding percent for each absorbance value, then transformed to concentration using the model fit values. Samples were re-measured or excluded (n = 3) if the CV between replicates exceeded 20% or the value lay outside of the stable range of the standard curve (20-80% tracer binding). Samples were initially run at a 200X dilution and re-analyzed at a higher dilution if necessary when samples were not on the standard curve. The detection limit for each plate was calculated at 80% tracer binding. Samples above 80% binding were deemed below the limit of detection and could not yield reliable concentrations.

Standard concentrations of IGF-1 were compared across plates and individual replicates were excluded if they fell outside of the interquartile range for each standard. Inter-assay pools (IPs) at four concentrations (low, medium, medium-high, and high) were included on each plate to account for inter-assay variability. The IP concentrations were compared across plates, and a correction factor was applied to plate values where IPs fell outside the interquartile range. Corrections were made by running a linear regression between the IP values on individual plates and the average IP values across all plates. Sample concentrations were normalized using the slope and intercept from this regression.

2.7 RNA extraction and Sequencing

Homogenization of gill and liver tissue and total RNA extraction were performed following previously established methods[^70^](https://paperpile.com/c/Ab0ybX/fOQqw) with some modifications. Briefly, gill and liver tissues were homogenized in TRIzol (Ambion, Foster City, CA, USA) and BCP reagent (Sigma-Aldrich, Oakville, ON, Canada) using stainless steel beads and a MM301 mixer mill (Retsch, Haan, Germany). Next, a ‘No - Spin Procedure’ was used to extract RNA from the homogenized tissue using the MagMAX-96 total RNA isolation kit (Ambion) on a Biomek FXP automation workstation (Beckman Coulter, Mississauga, ON, Canada). Following extraction, gill and liver RNA purity and quantity was assessed using an infinite M nano+ 200 pro (Tecan, Austria).

From the final food deprivation timepoint, 15 gill and liver samples were selected from each temperature and feeding treatment (n = 120 samples total, 60 per tissue) for sequencing based on their RNA quality and integrity. Total RNA integrity of selected samples was measured using a BioAnalyzer 2100 (Agilent) and samples with strong integrity were then shipped to Canada's Michael Smith Genome Sciences Centre (Vancouver, BC, Canada) for cDNA library preparation and sequencing.

Samples were sequenced on a single Novaseq 10B flow cell on the Novaseq X plus (Illumina) producing approximately 3.5 billion total reads with an average of 29 million ( ± 2.5 million SD) reads per sample. Upon arrival at the sequencing center, the quality of each RNA sample was reassessed using an Agilent Bioanalyzer RNA Nanochip (Thermo Fisher Scientific), and then polyadenylated (PolyA+) RNA was purified using the NEBNext Poly(A) mRNA Magnetic Isolation Module (E7490L, New England Biolabs) from 500 ng total extracted RNA. Poly(a) selection to isolate mRNA from total RNA, was performed using NEBNext Oligo d(T)_25_ beads incubated at 65^o^C for 5 min followed by snap-chilling at 4^o^C to denature RNA and facilitate binding of poly(A) mRNA to the beads. Elution of mRNA was then conducted placing the beads in Tris Buffer, incubated at 80^o^C for 2 min then held at 25^o^C for 2 min. RNA binding buffer was added to allow the mRNA to re-bind to the beads. This was then thoroughly mixed by pipetting the solution up and down 10 times and incubated at room temperature for 5 min before removing the supernatant and washing the mRNA bound beads. Finally the mRNA was eluted from the beads in 20 µL Tris buffer incubated at 80^o^C for 2 min.

 First-strand cDNA was then synthesized from heat-denatured purified mRNA using a Maxima H Minus First Strand cDNA Synthesis kit (Thermo-Fisher, USA) and random hexamer primers at a concentration of 200 ng.µL^-1^ along with a final concentration of 40 ng.µL^-1^ Actinomycin D. This was followed by PCR Clean DX (Aline Biosciences) bead purification on a Microlab NIMBUS robot (Hamilton Robotics, USA). Second strand cDNA was then synthesized following the NEBNext Ultra Directional Second Strand cDNA Synthesis protocol (New England Biolabs).

 The produced cDNA was then fragmented by Covaris LE220 sonication to achieve 250-300 bp average fragment lengths. The sheared cDNA was subject to end-repair and phosphorylation in a single reaction using an enzyme premix (New England Biolabs) containing T4 DNA polymerase, Klenow DNA Polymerase and T4 polynucleotide kinase, incubated at 20^o^C for 30 min. Repaired cDNA was purified in 96-well format using PCR Clean DX beads (Aline Biosciences, USA), and 3’ A-tailed (adenylated) using Klenow fragment (3’ to 5’ exo minus) followed by incubation at 37^o^C for 30 min prior to enzyme heat inactivation. Illumina PE adapters were ligated at 20^o^C for 15 min and then the adapter-ligated products were purified using PCR Clean DX beads, digested with USER^TM^ enzyme (1U.µL^-1^, NEB) at 37^o^C for 15 min, and immediately run for 10 cycles of indexed PCR using NEBNext Ultra II Q5 DNA Polymerase (New England Biolabs) and Illumina’s PE primer set. PCR parameters: 98˚C for 1 min followed by 10 cycles of 98˚C  15 s, 65˚C  30 s and 72˚C  30 s, and then 72˚C  5 min. The PCR products were purified and size-selected using a 1:1 PCR Clean DX beads-to-sample ratio (twice), and the eluted DNA quality was assessed with Caliper LabChip GX for DNA samples using the High Sensitivity Assay (PerkinElmer, Inc. USA) and quantified using a Quant-iT dsDNA High Sensitivity Assay Kit on a Qubit fluorometer (Invitrogen) prior to library pooling and size-corrected final molar concentration calculation for Illumina sequencing with paired-end 150 base reads.

2.9 Differential Expression and Functional Enrichment

To assess between fed and food deprived individuals bioinformatic approaches were used to identify differentially expressed transcripts and evaluate the processes that these transcripts are involved in for both gill and liver tissue.

Each transcript was first counted in STAR-produced aligned read files using featureCounts v2.0.6 with default settings[^93^](https://paperpile.com/c/Ab0ybX/23qpU) to produce a number of counts for each transcript that could be used for relative quantification. Total counts were then assessed for differential expression using R v.4.2.1 package EdgeR v.3.38.4[^94,95^](https://paperpile.com/c/Ab0ybX/wwqfH+ibfGY). Prior to analysis, the “filterByExpr” function was used, with default arguments, to retain transcripts for which expression was detected across all individuals, ultimately resulting in a final count of 28,370 gill transcripts and 21,357 liver transcripts. Those resulting gene-expression sequence data were analysed via multidimensional scaling (MDS) analysis, conducted independently for each tissue using the number of counts for each of the above 28,370 transcripts from the gill and 21,357 transcripts from the liver, using the “plotMDS” function in the limma package[^96^](https://paperpile.com/c/Ab0ybX/lSU5D).

Next, transcripts differentially expressed between fed and food-deprived individuals within the same temperature were tabulated using two different expression levels: (A) all differentially expressed transcripts with an absolute value log_2_ Fold Change (log_2_FC) > 0 (representing all significantly differentially expressed transcripts) and (B) highly differentially expressed transcripts with an absolute value log_2_FC > 2, a threshold chosen to select for transcripts which were four times as highly expressed (Figure 2). All differentially expressed transcripts were assessed to evaluate overall transcriptional changes induced by food deprivation in the liver and gill, while highly differentially expressed transcripts were focused on for further functional analysis and biomarker development. A *q*-value filter of < 0.05 (False discovery rate adjusted p-value) was applied to all differentially expressed transcripts[^97^](https://paperpile.com/c/Ab0ybX/5e9bO). Log_2_ counts per million (CPM) data for all 28,370 transcripts from the gill and 21,357 transcripts from the liver were then generated and are available in Supplemental File 2.

Finally, transcripts conserved across temperature treatments were identified, annotated, and queried against gene ontology databases to identify functional processes impacted by food deprivation. Using the lists of highly differentially expressed transcripts (those > 2 log_2_FC) between fed and food-deprived individuals, conserved transcripts which were differentially expressed in food-deprived individuals from both temperature treatments were identified independently in both the gill and liver. The lists of highly differentially expressed transcripts that were conserved between temperature treatments were then annotated to the Chinook salmon genome[^90^](https://paperpile.com/c/Ab0ybX/2JcTJ) and conserved molecular processes that were modified by food deprivation were identified using functional enrichment performed using gene ontology (GO) terms through enrichR[^98–100^](https://paperpile.com/c/Ab0ybX/N9dA5+6xE1m+Gkt68). The transcripts were queried against the “GO_Biological_Processes_2021”, “GO_Molecular_Function_2021”, “GO_Cellular_Component_2021” and “KEGG_2016” databases, with positive hits returned representing an adjusted p-value < 0.05 (the null hypothesis for this test is that the distribution of transcripts representing a particular GO term is the same between the differentially expressed transcripts and background transcript sets[^101^](https://paperpile.com/c/Ab0ybX/b3n4v); All functional enrichment output available in Supplemental File 3). Finally, the unique positive hits from the GO enrichment database (GO terms) in the GO Biological Process category were identified using REVIGO (Supek et al., 2011) for summary and visualization for both the gill (Supplemental Figure 5) and liver (Supplemental Figure 6) independently. Adjusted p values were supplied for all REVIGO analysis to guide the clustering of redundant GO terms by the REVIGO algorithm[^102^](https://paperpile.com/c/Ab0ybX/X7Fp9). All REVIGO output is available in Supplemental File 4.

2.10 Biomarker Identification

Statistical analyses were then applied to evaluate the relative expression of the 19 candidate biomarkers across treatment groups as well as in relation to the physiology of each fish. A PCA was applied to the CPM values of the final 19 biomarkers using the factomineR[^100^](https://paperpile.com/c/Ab0ybX/pEkJJ) and factoextra[^101^](https://paperpile.com/c/Ab0ybX/juH2f) packages in R to determine if there was separation between fed and unfed treatments at 8^o^C and 16^o^C. Finally, Spearman’s correlations was used to investigate the relationship between the CPM transcript abundance of each candidate biomarker with physiological metrics of hepatosomatic index, condition factor, muscle energy density and plasma IGF-1 concentration at the end of food deprivation, using the corr package.

2.11 Biomarker Assay Development, Testing and Application

TaqMan assays were developed targeting each of the 19 candidate biomarkers discovered in gill tissue through the biomarker identification process. Two assays for each candidate biomarker were then tested across five series serial dilutions of RNA from 8 salmonid species (Chinook salmon, coho salmon *O. kisutch*, chum salmon *O. keta*, pink salmon *O. gorbuscha*, sockeye salmon *O. nerka*, Atlantic salmon *Salmo salar*, brown trout *Salmo trutta*, and arctic char *Salvelinus alpinus*) to evaluate primer efficiency (calculated as efficiency(%) = (10^-1/slope^-1) x 100) and select the best assays for application (Table 1).

Table 1. TaqMan assays and PCR efficiencies of candidate biomarkers across 8 salmonid species

See Table 1 Excel File

Following biomarker testing, efficient primer and assay sets for the 19 candidate biomarkers were sequentially run on a total of 585 juvenile Chinook salmon which consisted of all samples from every fish throughout the food deprivation and refeeding portion of the experiment. Total RNA was extracted from the gill tissue of each fish as described above and cDNA was synthesized from 62.5 ng.ml^-1^ total RNA using SuperScript VILO synthesis kits (Invitrogen) as previously described[^49,70^](https://paperpile.com/c/Ab0ybX/oZIOS+fOQqw), with negative controls for cDNA synthesis including no reverse transcriptase (no RT), no RNA, and cDNA. Using the microfluidics Fluidigm Biomark HT platform, gene expression levels were assayed for the 19 curated biomarkers discovered during biomarker identification and three reference genes (*glul*, *fkbp10* and *hla21*; all used as consistently expressed “housekeeping” genes that were determined to be the most stable using Normfinder[^106^](https://paperpile.com/c/Ab0ybX/v9gQC)) . Prior to full 96.96 dynamic array fluidic circuit[^71^](https://paperpile.com/c/Ab0ybX/RTQtW) runs, specific target amplification was performed using a pool of low concentrations of the primer pairs (0.2 μM of each of the primers), 3.76 μL 1× TaqMan PreAmp master mix (Applied Biosystems), and 1.24 μL of cDNA, run on a 14 cycle PCR program as previously described[^49,70^](https://paperpile.com/c/Ab0ybX/oZIOS+fOQqw) to ensure an adequate abundance of target for each primer set. Following an ExoSap-IT (Applied Biosystems) clean up step to remove any residual primers, amplified samples as well as primers and probes were run on eight 96.96 gene expression dynamic arrays (Standard Biotools Corporation, CA, USA) following the Biomark platform instructions[^49,70,71^](https://paperpile.com/c/Ab0ybX/RTQtW+oZIOS+fOQqw). Included with samples on each dynamic array were serial dilutions (1/5, 1/25, 1/125, 1/625, 1/3125) of a cDNA pool of all the Chinook salmon samples to calculate the PCR efficiencies for each assay per chip. The pooled dilution series were run in each chip as the calibrator for the 2^-ΔΔCt^ method and to control for chip to chip variation in GenEx 7.0 software (MultiD Analyses AB, Sweden).

Assays were run and scored via standard procedures for the microfluidics Fluidigm Biomark HT platform[^49,69,71^](https://paperpile.com/c/Ab0ybX/RTQtW+oZIOS+WvtEX). Each sample well was loaded with 3.0 µL 2 x Taqman master mix (Thermo Fisher Scientific, Ottawa, Ontario, Canada), 0.3 µL 20 x GE sample loading reagent (Fluidigm Biomark), and 2.7 µL STA product. Each assay well was loaded with 3.3 µL 2x assay loading reagent (Fluidigm Biomark), 0.7 µL DNA suspension buffer, 1.08 µL forward and reverse primers (50 μM), and 1.2 µL probe (10 μM). Cycling conditions were 50^o^C for 2 min, 95^o^C for 10 min, followed by 40 cycles of 95^o^C for 15 s and 60^o^C for 1 min. The qPCR data were then extracted using Real-Time PCR Analysis Software (Fluidigm Biomark) and Ct thresholds were manually scored for each assay across all chip runs. Primer efficiency for each assay on each chip was calculated using (10^1/slope^- 1) x 100, where the slope was estimated by plotting the Ct value for each assay over the serial dilutions of the cDNA. Then the expression of the samples for each chip run were normalized to the geometric mean expression values of *glul*, *fkbp10,* and *hla21* using the 2^-ΔΔCt^ method[^107^](https://paperpile.com/c/Ab0ybX/WGSwO). Before normalization, the assays that did not consistently amplify across all chips (*spryd3*), and samples with extreme outliers (> 3 Ct values compared to the pool sample) across all assayed transcripts (26 samples across all chips) were removed.

2.12 Data exploration and classification

The performance parameters including sensitivity, specificity and prediction accuracy were calculated as described by Akbarzadeh et al[^105^](https://paperpile.com/c/Ab0ybX/Kb6aS) and these metrics were assessed on the testing set. Sensitivity is the ratio of the true positives to true positives and false negatives combined, specificity is the ratio of true negatives to the true negatives and false positives combined, and prediction accuracy is the ratio of true positives plus true negative to the true positives plus true negatives plus false positive plus false negative. The latter calculates how a percentage of cases is correctly classified.

As the Salmon Fit-Chip has been developed to accommodate up to 12 biomarkers per classifier to be applied on wild salmon[^72,105,106^](https://paperpile.com/c/Ab0ybX/fOQqw+DlKu+Kb6aS), the final food deprivation classifier was optimized iteratively beginning with 18 biomarkers. In the RF-based classification model, the biomarkers for each classifier were ranked based on mean decrease in accuracy and Gini scores. The Gini score is a measure of variable importance that measures how classification accuracy is impacted when values for that variable are randomly permuted when classifying samples from the bootstrap test set[^104^](https://paperpile.com/c/Ab0ybX/tgnVp). Following each iteration of the RF classifier the lowest ranking biomarker by Gini score was removed until a panel comprising up to 12 biomarkers demonstrating the strongest set of the above random forest performance parameters was reached. In the process of further optimization, biomarker removal persisted until the point where the elimination of a single biomarker led to a drop in the above RF performance parameters, and the panel still consisted of both up- and down-regulated biomarkers. This process identified a panel with as few as 7 biomarkers that still resulted in the same random forest performance parameters as the full 12 biomarker panel.

The random forest classification model was built using the random forest R package which is based on the original algorithm of Breiman[^107^](https://paperpile.com/c/Ab0ybX/bvKGA). Finally, PCAs were used to plot the overall expression profiles of the 12 biomarkers throughout both food deprivation and refeeding highlighting the total separation and confidence intervals surrounding the expression of the 12 biomarkers.

2.13 ANOVA based Statistical Analysis

Body morphometrics and physiological data collected at the final food deprivation timepoint (condition factor, HSI, energy density, and IGF-1 concentration) were analyzed using two-factor ANOVA including temperature, treatment, and its interactions in the model as fixed effects. Physiological data collected throughout the full time series of food deprivation and refeeding (length, mass, condition factor, and HSI) were analyzed using two separate three-factor ANOVA, one focusing on the food deprivation portion of the experiment, with one focusing on the refeeding portion of the experiment. These three-factor ANOVA analyses included temperature, treatment, time and their interactions between the three factors in the model as main effects. The potential for tank effects was assessed by comparing AIC values for models both with and without a random effect of experimental tank for physiological metrics. As little to no tank effects were detected, tank effects were not included in the final models. For ANOVAs used to analyze physiological metrics, Shapiro-Wilk’s and Levene’s tests were used to assess normality of data and homogeneity of variance along with graphical investigations of fitted residual plots. If assumptions of either normality or homogeneity were violated, a ranked, log, or square root transformation was applied to the dataset.

For gene specific three-factor ANOVA during food deprivation and refeeding as well as two-factor ANOVA for candidate biomarkers at the end of food deprivation, models were constructed as described above and data was retained in its untransformed CPM or log_2_ value. To investigate potential false detections (fish classified as unfed in fed treatments), one-factor ANOVA were run on condition factor and hepatosomatic index to compare potential false detection fish with fed and unfed fish at the end of food deprivation. Following the evaluation of main and interactive effects, post hoc tests were performed with Tukey’s HSD test from the multcomp package[^108^](https://paperpile.com/c/Ab0ybX/Cfvlk). Full results of all ANOVA analysis can be found in Supplemental Table 2. All statistical analyses were performed using R 4.4.2 ([http://www.R-project.org/](http://www.r-project.org/)) with a significance level (α) of 0.05.

Supplemental Results:

3.3 Biomarker Identification, Training, and Testing

There were 12 unique gill biomarker candidates which were consistently downregulated at a log_2_FC > 2 and 7 unique candidates which were consistently upregulated at a log_2_FC > 1 conserved across both 8^o^C and 16^o^C temperatures in food deprived individuals when compared to their fed control counterparts. Biomarker candidates which were downregulated in food deprived individuals were associated with collagen formation (*col1a2, col9a1a, col9a2, col9a3, col10a1a, col2a1*), neuronal development (*cbln4, gatad2a*), microfibril formation (*mfap2*) and bone remodeling (*bglap*) consistent with overarching functional analysis (Supplemental Figure 7). Upregulated biomarker candidates exhibited more diverse functions and were associated with cellular differentiation *(klf15, egr1*), iron metabolism (*frrs1*), fatty acid oxidation (*acad11*), cytoskeletal organization (*spryd3*), immunoglobulin/connective tissue formation (*hmcn1*) and inflammation mediation (*cyp2j2*; Supplemental Figure 8).

A PCA including the counts per million (CPM) expression of all 19 candidate gill biomarkers demonstrated clear separation between fed and unfed treatments, with little impact of temperature (Figure 5A). Unfed and fed treatments primarily separated out linearly across PC1 (68% variance) while there was some minor, non-significant impact of temperature in fed treatments along PC2 (12% variance). Candidate biomarkers that contributed the most to the overall variation within the PCA were largely downregulated and composed of collagen forming units (*col9a2, col2a1, col9a1a, col10a1a;* Figure 5B). Further, there was one upregulated biomarker which contributed more than the default average expected value for the loadings of PC1 and PC2 (dotted red line in 5B) *frrs1* involved in iron transport, while other upregulated transcripts *klf15, cyp2j2, acad11, spryd3,* and *hmcn1* made similar but smaller contributions to observed variation.

The expression of candidate biomarkers was evaluated using high-throughput qPCR assays designed across salmonid species demonstrating strong efficiencies (80-120%) for most primer sets across the 8 salmonid species and their potential for cross-species application (Table 1). Next, using the process of iterative optimization, removing individual biomarkers and retraining the classifier to test its performance, we determined that reducing the total number of biomarkers from an initial set of 18 down to 7 did not impact the classification performance (Supplemental Table 3). Random forest classifiers trained on biomarker panels ranging from 12 (Figure 6A) to 7 biomarkers showed an area under the receiver operator characteristic curve of 100% on the training set (N = 76) demonstrating perfect sensitivity and specificity for classification of both fed and unfed treatments (Figure 6B).

On the testing dataset (N = 38), a random forest classification model trained on 12 to 7 biomarkers showed high prediction accuracy of 97.4% and sensitivities of 100% and 94.7% for unfed and fed control fish, respectively. However, when the classifier was reduced to 6 biomarkers, removing the final upregulated biomarker, classifier sensitivity dropped to 84% in fed control fish and prediction accuracy dropped to 94.7%, highlighting the importance of including upregulated biomarkers for classifier training and testing (Supplemental Table 3). Finally, we selected the top 12 biomarkers to make up the final classifier, aiming for a combination of both up-(*cyp2j2*, *frrs1*, *acad11*, and *klf15*) and down-regulated (*col1a2.v1, mfap2, bglap, col9a1a, col1a2.v2, col9a3, col10a1a,* and *col9a2*) genes, in unfed versus fed individuals regardless of environmental temperature (p < 0.05; Figure 6A). However it is noteworthy that a 7 biomarker panel using 1 upregulated biomarker (*cyp2j2*) and 6 downregulated biomarkers (*cola1a2.v1*, *mfap2, blgap, col9a1a, col1a2.v2* and *col9a3*) performed efficiently, with the same sensitivity and prediction accuracy as the 12 gene biomarker panel when applied to the testing dataset (Supplemental Table 3). All together, the strongest food deprivation biomarkers in the models were *col1a2v1*, *mfap2, bglap*, and *cola9a1a* demonstrating Gini scores greater than 5 while all other biomarkers fell below 3.5 (Figure 6C), and all four of these biomarkers were included in both the 12 and 7 biomarker versions of the random forest classification model.

3.4 Biomarker Detections Throughout Food Deprivation and Refeeding

By day 3 of refeeding 11 of 12 biomarkers remained differentially expressed between fed and unfed individuals in both 8^o^C and 16^o^C with the exception of *cyp2j2* (p < 0.05; Supplemental Figure 10). While *col9a2* returned to control levels of expression by day 6 in 8^o^C, the remaining biomarkers continued to be differentially expressed for 9-12 days (p < 0.05). In contrast, in 16^o^C most biomarkers returned to control levels of expression by 3 to 6 days of refeeding, while only *bglap* remained consistently differentially expressed until 9 days of refeeding (p < 0.05). By day 18 in 16^o^C, some biomarkers which were downregulated by food deprivation had increased their expression so much during recovery that they were increased when compared to control fish (*col1a2.v1, mfap2, col9a1a, cola1a2.v2,* and *col9a2*; p < 0.05). However, by the end of the refeeding period most biomarkers (with the exception of *col1a2.v1* in 8^o^C) were no longer differentially expressed between fed and unfed individuals.

Investigation of the overall expression profiles depicted by qPCR of the 12 optimal biomarkers contributing to food deprivation classification through PCAs (Sufpplemental Figures 10, 11) demonstrated clear separation between fed and unfed treatments. During food deprivation, unfed fish in 16^o^C demonstrated complete separation from their fed control counterparts through PCA, beginning at 14 days and this separation persisted throughout the remainder of food deprivation (Supplemental Figure 11B-D). Similarly, but delayed, unfed fish in 8^o^C demonstrated near total separation from their fed control counterparts beginning at day 28 with complete separation by day 49 (Supplemental Figure 11C-G). During days 3 and 6 of refeeding, fed and unfed treatments demonstrated some separation (Supplemental Figure 11A, B), however all treatments were strongly clustered together for the remainder of refeeding (Supplemental Figure 12C-G). The rebound and overexpression signature of *col1a2.v1, mfap2, col9a1a, cola1a2.v2,* and *col9a2* at day 18 in 16^o^C led to a higher degree of separation through PCA at day 18 of refeeding when compared to day 15 or day 21 (Supplemental Figure 12E-G). All data depicted by PCA both during food deprivation and refeeding is available in supplemental file 6.
