# Supplementary material for: Identification of food deprivation in salmonids using gill biomarkers
Source: Conserv Physiol. 2025 Dec 19;13(1):coaf088. doi: 10.1093/conphys/coaf088 (PMC12716027; doi:10.1093/conphys/coaf088)
Supplement: Web_Material_coaf088 [file web_material_coaf088.zip › Supplemental Table 3 Biomarker Panel Optimization.docx]

Supplementary Table 3. Optimization analysis to select the ultimate biomarkers for food deprivation classification analysis. The initial Random Forest-based model classification was applied on all the biomarkers using the training data to rank and select the ultimate biomarkers for final RF classification. Sensitivity scores were calculated for 1/3 of the testing data to demonstrate the performance of the Random Forest (RF) model. The expression direction of the biomarkers in response to food deprivation is also provided.

| Gene name | Regulation | RF Classifier - 18 | RF Classifier - 12  Final classifier | RF Classifier - 8 | RF Classifier - 7  Most efficient | RF Classifier - 6 |
| --- | --- | --- | --- | --- | --- | --- |
| col1a2v1 | Down | Rank 1 | Rank 1 | Rank 1 | Rank 2 | Rank 3 |
| mfap2 | Down | Rank 2 | Rank 2 | Rank 2 | Rank 4 | Rank 2 |
| bglap | Down | Rank 3 | Rank 3 | Rank 4 | Rank 1 | Rank 1 |
| col9a1a | Down | Rank 4 | Rank 4 | Rank 3 | Rank 3 | Rank 4 |
| col1a2v2 | Down | Rank 5 | Rank 5 | Rank 5 | Rank 5 | Rank 5 |
| col9a3 | Down | Rank 6 | Rank 6 | Rank 7 | Rank 6 | Rank 6 |
| cyp2j2 | Up | Rank 7 | Rank 7 | Rank 6 | Rank 7 |  |
| frrs1 | Up | Rank 8 | Rank 8 | Rank 8 |  |  |
| col10a1a | Down | Rank 9 | Rank 9 |  |  |  |
| col9a2 | Down | Rank 10 | Rank 10 |  |  |  |
| acad11 | Up | Rank 11 | Rank 11 |  |  |  |
| klf15 | Up | Rank 12 | Rank 12 |  |  |  |
| ndtdc | Down | Rank 13 |  |  |  |  |
| cbln4 | Down | Rank 14 |  |  |  |  |
| egr1 | Up | Rank 15 |  |  |  |  |
| hmcn1 | Up | Rank 16 |  |  |  |  |
| gatad2a | Down | Rank 17 |  |  |  |  |
| cenpp | Down | Rank 18 |  |  |  |  |
|  |  |  |  |  |  |  |
| Sensitivity for 1/3 testing data |  | 1-Unfed = 100.0%  2-Fed = 94.7% | 1-Unfed = 100.0%  2-Fed = 94.7% | 1-Unfed = 100.0%  2-Fed = 94.7% | 1-Unfed = 100.0%  2-Fed = 94.7% | 1-Unfed = 100.0%  2-Fed = 84.2% |
| Prediction accuracy for 1/3 testing data |  | 1-Unfed = 97.4%  2-Fed = 97.4% | 1-Unfed = 97.4%  2-Fed = 97.4% | 1-Unfed = 97.4%  2-Fed = 97.4% | 1-Unfed = 97.4%  2-Fed = 97.4% | 1-Unfed = 94.7%  2-Fed = 94.7% |
